# The fear of conflict leads people to systematically avoid potentially valuable zero-sum situations

**DOI:** 10.1038/s41598-022-22849-y

**Published:** 2022-10-26

**Authors:** Shai Davidai, Michael W. White, Genevieve Gregorich

**Affiliations:** Columbia Business School, Columbia University, New York, NY 10027 USA

**Keywords:** Psychology, Human behaviour

## Abstract

From interpersonal interactions to international arms races, game theorists and social scientists have long studied decision-making in zero-sum situations. Yet, what happens when people can freely choose whether to enter zero-sum situations in the first place? Thirteen studies (including five pre-registered) consistently document evidence for *zero-sum aversion—the desire to avoid situations that are (or are believed to be) zero-sum.* Across different contexts (economic games, market entry decisions, performance reviews, negotiations, job applications), samples (online participant pool, MBA students, community sample), and designs (within- and between-participant, real and hypothetical decisions), people avoid zero-sum situations that inversely link their and others’ outcomes as well as refrain from putting *others* in such situations. Because people fear that zero-sum situations will be rife with conflict, they exhibit zero-sum aversion even when doing so is costly. Finally, we find that people require zero-sum situations to provide substantially higher payoffs (e.g., compensation) to overcome their zero-sum aversion. We conclude with a discussion of the implications for interpersonal and intergroup conflict.

## Introduction

People often view interpersonal, intergroup, and international relations as zero-sum, seeing one party’s gains as offset by other parties’ losses^1–5^. This belief can have broad implications, eroding interpersonal trust and increasing hostility, prejudice, misogyny, and anti-immigrant sentiment^2,5–10^. Even when people try to resolve their differences, expecting diametrically opposed outcomes can lead them to overlook mutually beneficial agreements, harm their relationships, and “leave money on the table”^6,11,12^. Regardless of whether a situation is truly zero-sum, viewing it as such clearly aggravates relationships between people, groups, and nations.

Research has assumed that people must engage in zero-sum interactions and has therefore focused on how they do so. Yet, many real-life situations allow people to choose whether they wish to interact with others, who they wish to interact with, and what level of interaction they seek. By focusing on how people behave in zero-sum situations, research has neglected the possibility that people may avoid such interactions altogether. Just as people sometimes avoid potentially beneficial economic games^13–15^, we suggest that they more generally avoid any situation that inversely links their and others’ outcomes. Thus, we raise a critical question: What happens when people can choose whether they wish to enter zero-sum situations?

We suggest that, when given the option to do so, people exhibit *zero-sum aversion*—*the desire to avoid situations that are (or are believed to be) zero-sum*. Importantly, we argue that this aversion is exhibited even when doing so is costly and both when situations are *objectively* zero-sum and when people *subjectively* see them as such. Rather than focus on people's reactions to existing outcomes of allocation decisions, we focus on their aversion to the *process* of such allocations. Independent of an allocation’s outcome (i.e., whether people receive more, less, or same as others), we suggest that people prefer to avoid processes that inversely links their and others’ outcomes.

Why do people exhibit zero-sum aversion? We suggest that people avoid zero-sum situations because they fear that these situations will elicit conflict and animosity. Zero-sum beliefs often make aggressiveness seem appropriate^16–18^ and people may therefore view zero-sum situations in general as prone to conflict. Such fear of conflict may arise in dyadic interpersonal and intergroup relations (e.g., expecting zero-sum resource allocation to elicit conflict between two individuals, groups, or nations) as well as in multi-party dynamics (e.g., expecting zero-sum organizational procedures to elicit conflict among many coworkers). Since people often withdraw from situations where they expect conflict^15^, and since such expectations often predict the actual conflicts people end-up experiencing^19^, they are likely to take their fear of conflict into consideration and avoid situations that are (or are believed to be) zero-sum.

Take, for example, the case of negotiations—a context with much potential for conflict. Decades of research examined how zero-sum beliefs affect trust, cooperativeness, and the “bottom line” in negotiations^12^. Yet, people often choose whether (and with whom) they wish to negotiate, leaving open the question of how seeing negotiations as zero-sum affects their willingness to do so. Although the propensity to initiate negotiations varies by gender, race, and other individual differences^20–22^, it is unclear if it is also affected by whether the negotiation itself is zero-sum. Thus, viewing negotiations as zero-sum may not only affect how people behave in them, but also whether they choose to negotiate in the first place.

This, of course, may be true of any situation people see as zero-sum. People may decline job opportunities where compensation is high but based on relative performance, entrepreneurs may avoid lucrative industries that require competing for market share, warring countries may avoid peace talks over seemingly zero-sum issues, and so forth. Fearing that zero-sum situations will foster conflict may lead people to avoid them, choosing less lucrative jobs, business endeavors, or peaceful courses of action.

We examine zero-sum aversion across thirteen main studies and eleven additional studies in the Supplementary Materials, documenting this phenomenon across various contexts that share an underlying psychological structure. First, we examine the effect of *subjective zero-sum beliefs* (Study 1A) and *objective zero-sum situations* (Study 1B), finding consistent evidence of zero-sum aversion in both real life and in hypothetical negotiations. Following, we examine the generalizability of zero-sum aversion in various real-life and hypothetical situations (Studies 2A-2C), which we then examine in a sequence of economic games involving real monetary outcomes (Studies 3A and 3B). Next, we test whether the fear that zero-sum situations will involve more conflict leads to zero-sum aversion (Studies 4A and 4B), whether the fear of conflict reduces people’s willingness to put *other people* in zero-sum situations (Study 4C), and whether manipulating this fear reduces zero-sum aversion (Studies 5A and 5B). Finally, we examine a real-world consequence of zero-sum aversion—increased wage requirements—among a sample of professionals (Study 6).

## Results

### Studies 1A and 1B

We begin by documenting zero-sum aversion in negotiations, a potentially costly, real-world context. Although negotiations are rarely zero-sum^12^, people often see them as such^11,23,24^. Yet, whereas past research focused on how zero-sum beliefs affect behaviors *during* negotiations, we examine whether such beliefs lead people to avoid negotiations altogether. Specifically, we examine how *subjective beliefs* about negotiations (whether negotiations are seen as zero-sum; Study 1A) and their *objective structure* (whether negotiations are indeed zero-sum; Study 1B) affect people’s propensity to engage in them and whether such zero-sum aversion is exhibited beyond any general individual preference for prosocial resource allocation.

#### Study 1A

We measured the prevalence of zero-sum beliefs and the propensity to initiate negotiations among a cohort of MBA students (*N* = 205). Compared to those with lower zero-sum beliefs, participants high in zero-sum beliefs exhibited substantial zero-sum aversion—believing that negotiations create harmful conflict (*r*(205) = 0.271, *p* < 0.001) and feeling apprehensive about initiating them (*r*(205) = 0.206, *p* = 0.003). In contrast, these participants were not more prone to recognize situations as negotiable (*r*(205) = − 0.023, *p* = 0.74) and actually felt more entitled to better outcomes (*r*(205) = 0.171, *p* = 0.014), suggesting that zero-sum beliefs were *uniquely* related to the propensity to* initiate *negotiations. Moreover, this relationship was exhibited even when controlling for the Big-Five personality traits (*ß* = 0.198, *p* = 0.005; Tables S1 and S2 in the SOM). Finally, we found that the belief that negotiations create harmful conflict mediated the effect of zero-sum beliefs on this avoidance (indirect effect: *ß* = − 0.280, *SE* = 0.069; 95% CI[− 0.413, − 0.146]; direct effect: *ß* = 0.008, *SE* = 0.069; 95% CI[− 0.127,0.144]). The more participants saw interpersonal relationships as zero-sum, the more they worried that negotiations create harmful conflict and, consequently, the more reluctant they felt about initiating them.

#### Study 1B

In Study 1B (*N* = 202), participants imagined working at an organization undergoing restructuring negotiations and were told that two issues were under consideration: a (zero-sum) resource allocation and a (non-zero-sum) workflow integration. They then indicated their willingness to represent their department in each negotiation.

We predicted that participants would avoid negotiations involving zero-sum issues. Indeed, participants exhibited zero-sum aversion, preferring the non-zero-sum integrative negotiation (M = 4.78, SD = 1.69) over the zero-sum distributive negotiation (M = 3.72, SD = 1.90), matched-pairs *t*(199) = 5.48, *p* < 0.0001, *dz* = 0.39.

Next, we examined whether zero-sum aversion was exhibited beyond individual differences in Social Value Orientation—a measure of social preferences in resource allocation^25^. Although social preferences moderated the aversion for inversely linking one’s own and others’ outcomes (*F*(2,195) = 3.31, *p* = 0.039), participants exhibited zero-sum aversion both when they were personally prone to favor prosocial allocations (M_diff_ = 1.36, *t*(126) = 5.69, *p* < 0.0001, *dz* = 0.51) and when they were prone to favor individualistic allocations (M_diff_ = 0.72, *t*(63) = 5.69, *p* = 0.047, *dz* = 0.25). While individual differences in social preferences matter (i.e., prosocial-leaning people exhibit somewhat stronger zero-sum aversion), the avoidance of zero-sum *situations* is exhibited beyond any such preferences.

### Studies 2A, 2B, and 2C

We next examine the generalizability of zero-sum aversion in controlled lab experiments involving market entry decisions (Study 2A), performance reviews (Study 2B), and real-effort tasks (Study 2C). In all studies, participants chose between a zero-sum option and a non-zero-sum option. We predicted that participants would exhibit zero-sum aversion, choosing to separate their and others’ outcomes over an option that inversely links them (i.e., zero-sum).

#### Study 2A

Study 2A (*N* = 100) examines zero-sum aversion in market entry decisions. We test whether people prefer to start businesses that expand the market (i.e., non-zero-sum) rather than capture existing market share (i.e., zero-sum) and whether this occurs even when success is more likely in the zero-sum option. Participants imagined choosing a location for a new business (a restaurant) between two options varying on several dimensions, including their potential to expand the market, with one location offering limited expansion and another location offering the potential for market expansion.

We predicted that participants would prefer to start a business that requires market expansion rather than a business that requires capturing existing market share, even when doing so can impede their success. Indeed, participants exhibited zero-sum aversion, choosing a location where customers would come from expanding the market (M = 5.18, SD = 1.53) over a location where they would have more customers but would need to attract them from other businesses (M = 4.00, SD = 1.54), matched-pairs *t*(99) = 4.33, *p* < 0.001, *dz* = 0.43. Thus, participants overwhelmingly opted for the riskier non-zero-sum option, choosing lower chances of success over a situation where others’ outcomes would come at their expense (and vice-versa).

#### Study 2B

Participants in Study 2B (*N* = 105) read about a company with two types of performance reviews, both of which offer equal chances of favorable evaluations: evaluating employees relative to their colleagues (i.e., zero-sum) or relative to absolute criteria (i.e., non-zero-sum). We predicted that participants would be averse to the zero-sum process, where one employee’s success comes at other employees’ expense. Indeed, participants significantly preferred to be evaluated by the non-zero-sum review (M = 5.68, SD = 1.63) over the zero-sum review (M = 2.95, SD = 1.87), matched-pairs *t*(104) = 9.22, *p* < 0.001, *dz* = 0.96. A similar pattern was exhibited in a forced-choice measure: 82% of participants (n = 86) preferred a non-zero-sum review, but only 18% (n = 19) preferred a zero-sum review, *χ*^*2*^(1,104) = 46.27, *p* < 0.001. Although both options offered equal chance of success, participants exhibited zero-sum aversion, choosing a (non-zero-sum) review that evaluates them relative to given performance criteria over a (zero-sum) review that evaluates them relative to their colleagues’ performance.

#### Study 2C

Study 2C (*N* = 199) examines zero-sum aversion in a real-effort task. As part of a workplace simulation, participants played the role of an intern in a magazine. In the *Difficult Task* condition, their job was to count the number of characters and spaces in several long and grammatically complex phrases. In the *Simple Task* condition, they simply counted the number of words in a few short phrases. Following, participants learned about a follow-up session involving similar tasks which may qualify them for a bonus. Participants then indicated their preference between attending a session in which the bonus allocation process would be zero-sum (i.e., based on relative performance) or non-zero-sum (i.e., based on absolute performance).

Participants disproportionately opted for the session where their bonuses would be independent from others’ bonuses: They significantly chose the non-zero-sum option (74.9%, n = 149) over the zero-sum option (25.1%, n = 50; *χ*^*2*^(1, 197) = 49.25, *p* < 0.001). This was true in both the *Difficult Task* condition (non-zero-sum: 75.8%, n = 72, zero-sum: 24.2%, n = 23, *χ*^*2*^(1, 93) = 24.04, *p* < 0.001) and the *Simple Task* condition (non-zero-sum: 74%, n = 77, zero-sum: 26%, n = 27, *χ*^*2*^(1, 102) = 25.27, *p* < 0.001), suggesting that the aversion to zero-sum reward allocations was unaffected by beliefs about the odds of outdoing others (*χ*^*2*^(1, 197) = 0.08, *p* = 0.776). Even when they thought they could complete the task faster and more accurately, participants chose to separate their and others’ outcomes.

### Studies 3A and 3B

Observing behaviors in controlled environments that model critical features of real-life situations is considered the “gold standard” for documenting robust psychological phenomena. Thus, Studies 3A and 3B examine zero-sum aversion in economic games involving real financial outcomes. Based on two pilot studies (Supplemental Studies S1 and S2 in the SOM), we gave participants a choice between two lotteries—one in which their payoffs would be inversely related to their counterpart’s payoffs (i.e., zero-sum) and one in which their payoffs would be independent from them (i.e., non-zero-sum). We predicted that participants would choose to bear real financial costs to avoid zero-sum situations.

#### Study 3A

In Study 3A (*N* = 207), participants were randomly assigned to play for either $1 or $5 and made a series of decisions between two lotteries with real monetary outcomes: a *non-zero-sum* option and a *zero-sum* option. In the *non-zero-sum* option, each player would enter a separate lottery, with 50% chance of gaining or losing $1 or $5 (depending on condition), guaranteeing that players’ payoffs are independent from each other. In the *zero-sum* option, both players would enter the same lottery, with 50% chance of gaining $1 or $5 (depending on condition) while their counterpart loses that same amount of money and 50% chance of losing $1 or $5 while their counterpart gains that money.

At this point, the experiment ended for participants who chose the zero-sum lottery. Participants who chose the non-zero-sum lottery decided between two additional options: the same *non-zero-sum* option from before (independently giving each player 50% chance of gaining or losing) and a new *zero-sum* option with better odds of winning (giving participants 60% chance of gaining while their counterpart loses and 40% chance of losing while their counterpart gains). Although this zero-sum option inversely links the players’ payoffs, it also offers participants better chances of winning than losing.

We repeated this procedure three times. Each time, participants who chose the safer zero-sum option ended the experiment. Those who chose the riskier non-zero-sum option saw two additional lotteries: the same *non-zero-sum* option (giving each player 50% chance of gaining or losing) and a new *zero-sum* option with even better odds of winning. This zero-sum option was, in sequence, a lottery with 70% chance of gaining (while their counterpart loses) and 30% chance of losing (while their counterpart gains), a lottery with 80% chance of gaining (while their counterpart loses) and 20% chance of losing (while their counterpart gains), and a lottery with 90% chance of gaining (while their counterpart loses) and 10% chance of losing (while their counterpart gains). The experiment ended after the 5th round.

Table 1 presents the results. First, we examined the percentage of participants who, in the first decision, chose the non-zero-sum option over the zero-sum option. Although both lotteries initially offered equal chance of winning, and despite playing for real money, participants were substantially more likely to choose the *non-zero-sum* lottery (78%, n = 161) over the *zero-sum* lottery (22%, n = 46), *χ*^*2*^(1,206) = 67.66, *p* < 0.001.

**Table 1:** The likelihood of choosing the zero-sum option across the five different stages in the $1 condition (left), $5 condition (middle) and both conditions combined (left) (Study 3A).

	$1 Condition			$5 Condition			Overall		
	% non-zero-sum	% zero-sum	χ^2^ test	% non-zero-sum	% zero-sum	χ^2^ test	% non-zero-sum	% zero-sum	χ^2^ test
1st choice	80.40	19.60	*n* = *102*	75.20	24.80	*n* = *105*	77.80	22.20	*n* = *207*
			*χ*^*2*^ = 40.4, *p* < 0.001			*χ*^*2*^ = 28.0, *p* < 0.001			*χ*^*2*^ = 67.7, *p* < 0.001
2nd choice	71.60	23.40	*n* = *81*	75.90	24.10	*n* = *79*	73.70	26.30	*n* = *160*
			*χ*^*2*^ = 15.6, *p* < 0.001			*χ*^*2*^ = 22.3, *p* < 0.001			*χ*^*2*^ = 37.6, *p* < 0.001
3rd choice	67.20	32.80	*n* = *58*	65	35	*n* = *60*	66.10	33.90	*n* = *118*
			*χ*^*2*^ = 7.0, *p* = 0.009			*χ*^*2*^ = 5.5, *p* = 0.019			*χ*^*2*^ = 12.5, *p* < 0.001
4th choice	78.40	21.60	*n* = *37*	67.70	33.30	*n* = *39*	72.40	27.60	*n* = *76*
			*χ*^*2*^ = 12.7, *p* < 0.001			*χ*^*2*^ = 4.4, *p* = 0.036			*χ*^*2*^ = 15.8, *p* < 0.001
5th choice	89.30	10.70	*n* = *28*	88.50	11.50	*n* = *26*	88.90	11.10	*n* = *54*
			*χ*^*2*^ = 19.7, *p* < 0.001			*χ*^*2*^ = 17.4, *p* < 0.001			*χ*^*2*^ = 37.2, *p* < 0.001

Of the 161 participants who chose the non-zero-sum lottery in the first choice, 74% (n = 118) still opted for it in the second choice rather than the zero-sum option with 10% better odds of winning, *χ*^*2*^(1,160) = 37.60, *p* < 0.001. Of these, 66% (n = 78) still chose the non-zero-sum lottery in the third choice rather than the zero-sum lottery that offered 20% better odds, *χ*^*2*^(1,117) = 12.46, *p* < 0.001. Of these 78 participants, 72% (n = 55) still chose the non-zero-sum lottery in the fourth choice rather than the zero-sum lottery that offered 30% better odds, *χ*^*2*^(1,75) = 15.76, *p* < 0.001. Finally, of the 55 participants who chose the non-zero-sum option in the previous four choices, 89% (n = 48) still preferred this lottery in the fifth (and final) choice over the zero-sum lottery that offered *40% better odds* of winning, *χ*^*2*^(1,53) = 37.19, *p* < 0.001. Thus, participants exhibited zero-sum aversion even when winning the zero-sum lottery was 10%, 20%, 30% and even 40% more likely. On average, participants needed 19.9% better odds of winning to switch from the non-zero-sum lottery to the zero-sum lottery (a conservative estimate, treating participants who chose the non-zero-sum lottery in the final choice as likely to have switched to the zero-sum lottery if these chances rose by an additional 0.1%).

#### Study 3B

Participants in Study 3A made sequential choices, which may prompt strategic decision making such as attempting to be (or appear) consistent. To rule this out, we conducted a preregistered replication (*N* = 506) in which participants made a single choice between two lotteries: a non-zero-sum lottery and a zero-sum lottery with varying chances of winning.

Participants chose between a *non-zero-sum* lottery and a *zero-sum* lottery in one of five randomly assigned conditions. The *non-zero-sum* option was the same across all conditions, entering each player into a separate lottery with 50% chance of independently gaining or losing $1. The *zero-sum* option, in which both players would enter the same lottery with inversely related payoffs, varied across the five conditions, randomly assigning participants to either the *50/50, 60/40, 70/30, 80/20, or 90/10* zero-sum options from Study 3A.

**Figure 1:**
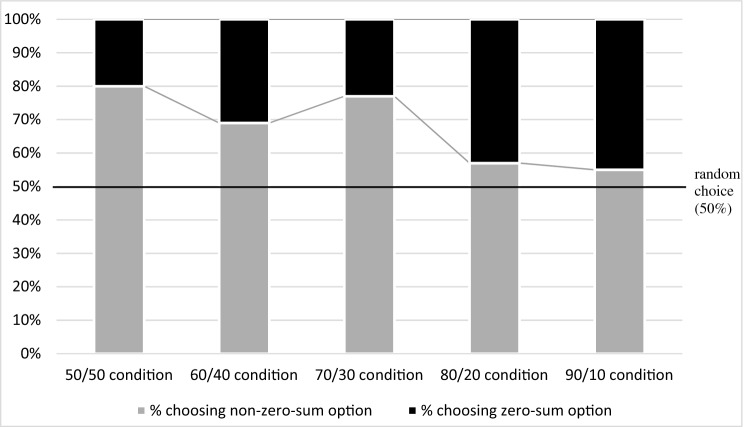
The likelihood of choosing the zero-sum option in each condition (Study 3B).

Figure 1 displays the results. As predicted, participants chose the *non-zero-sum* lottery significantly more often than the *zero-sum* lottery in the *50/50 condition* (zero-sum: 20%, n = 17, non-zero-sum: 80%, n = 67, *χ*^*2*^(1,83) = 31.83, *p* < 0.001), the *60/40 condition* (zero-sum: 31%, n = 34, non-zero-sum: 69%, n = 76, *χ*^*2*^(1,109) = 16.45, *p* < 0.001), and the *70/30 condition* (zero-sum: 23%, n = 21, non-zero-sum: 77%, n = 72, *χ*^*2*^(1,92) = 19.57, *p* < 0.001). Participants were also directionally more likely to choose the *non-zero-sum* lottery in the *80/20 condition* (zero-sum: 43%, n = 47, non-zero-sum: 57%, n = 63, *χ*^*2*^(1,109) = 2.34, *p* = 0.127) and the *90/10 condition* (zero-sum: 45%, n = 49, non-zero-sum: 55%, n = 60, *χ*^*2*^(1,108) = 1.12, *p* = 0.292). Although the zero-sum option offered equal or better odds at winning, and despite making only one choice in each condition, participants were quite averse to choosing it.

### Studies 4A–4C

Given the results of a pilot study (Supplemental Study S4 in the SOM), we next examine whether the fear of conflict lies at the heart of zero-sum aversion.

#### Study 4A

Participants (*N* = 101) were randomly assigned to read about a company that conducts performance reviews in a zero-sum or non-zero-sum manner (see Study 2A). Replicating our findings in a between-participant design, participants were significantly more averse to the zero-sum review process (M = 2.67, SD = 1.80, 95% CI[2.12, 3.23]) than the non-zero-sum process (M = 4.52, SD = 2.10, 95% CI[3.98, 5.06]), *t*(99) = 4.73, *p* < 0.001, *d* = 0.94.

Next, we examined expectations of conflict. As predicted, participants feared they will experience more conflict with their colleagues in the *zero-sum condition* (M = 5.90, SD = 1.14, 95% CI[5.47, 6.33]) than the *non-zero-sum condition* (M = 3.59, SD = 1.82, 95% CI[3.17, 4.01]), *t*(86.20) = 7.69, *p* < 001, *d* = 1.51, believing that inversely linking employees’ outcomes creates more tension and animosity than evaluating each employee independently.

**Figure 2:**
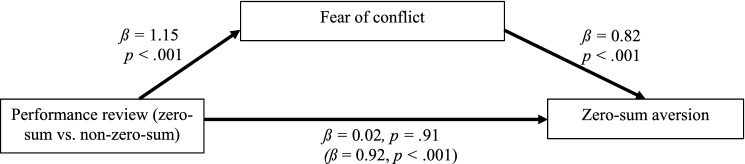
The mediating role of the fear of conflict on the relationship between type of performance review (zero-sum vs. non-zero-sum) and zero-sum aversion (Study 4A).

Finally, we examined whether the fear of conflict explained zero-sum aversion. Indeed, we found an indirect effect of condition on the preference for non-zero-sum procedures through conflict expectations, *ß* = − 0.94, 95% CI[− 1.31, − 0.62] and an insignificant direct effect of condition, *ß* = 0.02, 95% CI[− 0.36, 0.40] (Fig. 2). Thus, participants feared that zero-sum reviews will foster more conflict among colleagues and, consequently, were averse to them.

#### Study 4B

Study 4B conceptually replicates Study 4A in a new domain. Participants (*N* = 251) were randomly assigned to read about an organization undergoing restructuring negotiations, were told that the issue was either zero-sum (resource allocation) or non-zero-sum (workflow integration), and indicated their willingness to participate in them (see Study 1B).

First, we examined whether participants exhibited zero-sum aversion. Indeed, participants were less willing to represent their division in the negotiations when the issue was zero-sum (M = 3.12, SD = 1.84, 95% CI[2.80, 3.44]) rather than non-zero-sum (M = 3.81, SD = 2.00, 95% CI[3.46, 4.17]), *t*(249) = 2.88, *p* = 0.004, *d* = 0.65.

Next, we examined whether participants saw the zero-sum negotiations as more conflict prone. Participants feared conflict more in the *zero-sum condition* (M = 6.00, SD = 0.95, 95% CI[5.83, 6.17]) than the *non-zero-sum condition* (M = 4.84, SD = 1.17, 95% CI[4.63, 5.05]), *t*(249) = 8.62, *p* < 0.001, *d* = 1.09, viewing negotiations about zero-sum issues as fostering more conflict and animosity than negotiations about non-zero-sum issues.

Finally, we examined whether the fear of conflict mediated the effect of negotiation type on zero-sum aversion. Indeed, a bootstrapping mediation analysis (5,000 samples) revealed an indirect effect of condition on the willingness to negotiate through conflict expectations, *ß* = -0.66, 95% CI [-0.95, -0.39] and an insignificant direct effect, *ß* = -0.04, 95% CI [-0.56, 0.48]. Relative to participants who read about a non-zero-sum negotiation, those who read about a zero-sum negotiation expected it to spark more conflict and were therefore less willing to engage in it (Fig. S1 in the SOM).

#### Study 4C

Study 4C replicates and extends our findings by examining whether the fear of conflict fosters zero-sum aversion *even when making decisions for other people*. Participants (*N* = 251) imagined they were managers at a company who were looking to implement a new system for evaluating employees. As before, participants were randomly assigned to read about an evaluation system that conducts performance reviews in either a zero-sum or a non-zero-sum manner and indicated their preference for how to evaluate their employees.

Even when making decisions that would only affect other people (but not themselves), participants exhibited zero-sum aversion, showing a lower preference for a zero-sum review of their employees (M = 2.22, SD = 1.64, 95% CI[1.94, 2.51]) than a non-zero-sum review process (M = 4.31, SD = 1.97, 95% CI[3.97, 4.65]), *t*(249) = 9.11, *p* < 0.001, *d* = 1.15. Thus, exhibiting the robustness of the explored phenomenon, participants were averse to putting *other people* in zero-sum situations that negatively link their outcomes to each other.

We next examined expectations of conflict. As predicted, participants feared that their employees would experience more conflict in the *zero-sum condition* (M = 6.12, SD = 1.12, 95% CI[5.92, 6.31]) than the *non-zero-sum condition* (M = 3.74, SD = 1.85, 95% CI[3.42, 4.06]), *t*(249) = 12.31, *p* < 001, *d* = 1.55, believing that inversely linking  their employees’ outcomes would create more conflict than evaluating each employee independently.

Finally, we examined whether the fear of conflict explained participants’ zero-sum aversion. A bootstrapping mediation analysis (5,000 samples) revealed an indirect effect of condition on the willingness to implement a review system through conflict expectations, *ß* = − 1.53, 95% CI [− 2.01, − 1.10] and an insignificant direct effect, *ß* = − 0.56, 95% CI [− 1.16, 0.05]. That is, because participants feared that zero-sum reviews would foster conflict among their employees, they were less willing to implement such a system (Fig. S2 in the SOM).

### Studies 5A and 5B

Studies 5A and 5B examine whether concerns about conflict causally increase zero-sum aversion. Using two different manipulations, we examined whether alleviating the fear of conflict eliminates participants’ aversion to zero-sum situations.

#### Study 5A

Participants (*N* = 201) read the materials from Study 1B about negotiations involving resource allocation (zero-sum) and workflow integration (non-zero-sum) and were randomly assigned to one of two conditions. In the *Fear of Conflict* condition, they read that the company’s employees care deeply about their workplace relations and that helping one’s department is important but “not important enough to ruin your personal relationships.” In the *No Fear* condition, participants read that employees care about helping their departments “even at the cost of getting in conflict with others.” Participants in both conditions then indicated their willingness to participate in each of the two negotiations. If zero-sum aversion is due to a fear of conflict, then reducing such fear should reduce this aversion.

**Figure 3:**
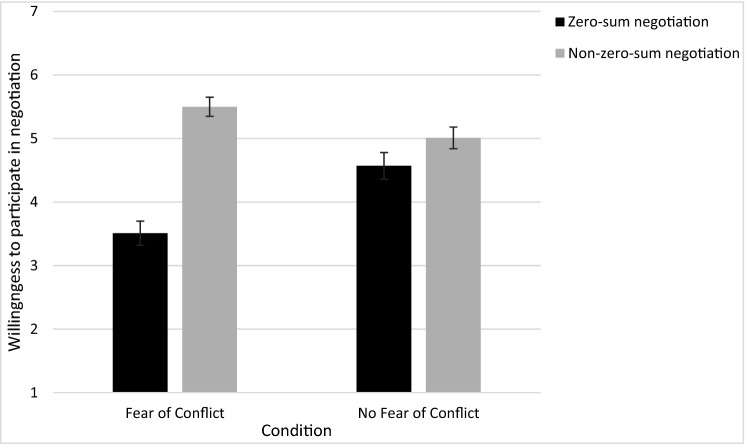
Participants' willingness to participate in a zero-sum and a non-zero-sum negotiation as a function of their fear of conflict (Study 5A). Errors bars represent standard errors (SEM).

As before, participants preferred the non-zero-sum negotiation (M = 5.25, SD = 1.64) over the zero-sum negotiation (M = 3.94, SD = 2.01), matched-pairs *t*(200) = 5.96, *p* < 0.001, *dz* = 0.42. However, this aversion was significantly moderated by concerns about conflict, *F*(1,199) = 9.66, *p* = 0.0012. A post-hoc Tukey test found that participants overwhelmingly preferred the non-zero-sum negotiation in the *Fear of Conflict* condition (M_non-zero-sum_ = 5.50, SD = 1.51; M _zero-sum_ = 3.51, SD = 1.87), *t* = 6.48, *p* < 0.001, but not in the *No Fear* condition (M_non-zero-sum_ = 5.01, SD = 1.74; M_zero-sum_ = 4.37, SD = 2.07), *t* = 2.11, *p* = 0.155 (Fig. 3). Thus, framing conflict as something that should not be feared eliminated participants’ aversion to zero-sum negotiations.

#### Study 5B

Study 5B further examines how concern about conflict fosters zero-sum aversion using a new manipulation of fear of conflict and a new measure of zero-sum aversion. Participants (*N* = 202) read about the two negotiations (involving a zero-sum issue or a non-zero-sum issue) and indicated which negotiation they would choose to join. Prior to making their decision, participants were randomly assigned to one of two conditions. In the *Fear of Conflict* condition, participants thought about which negotiation they would choose if they were extremely concerned about how the negotiations could lead to conflict and about the impact on their work relationships. In the *No Fear* condition, participants thought about which negotiation they would join were they not at all concerned about conflict or the impact on their relationships. Finally, participants indicated (in a trinary choice, including a ‘No Preference’ option) their preferred negotiation.

**Figure 4:**
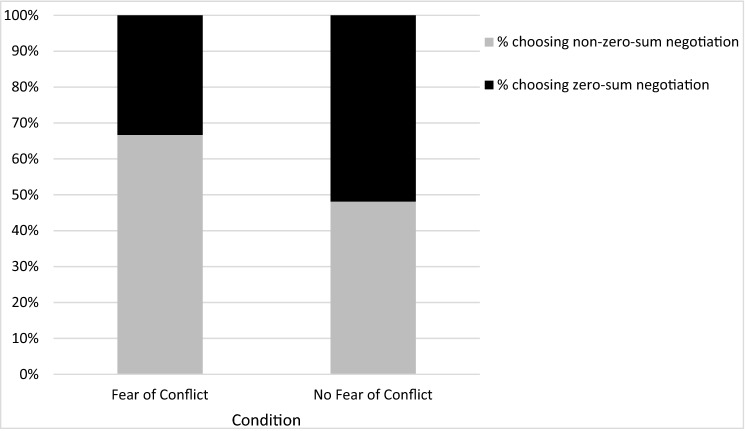
Participants' choice to join a zero-sum and a non-zero-sum negotiation as a function of their concern about conflict (Study 5B).

Replicating Study 5A with a new measure and a new manipulation, we found that the fear of conflict fostered zero-sum aversion, *χ*^*2*^(1, 163) = 5.75, *p* = 0.017. As shown in Fig. 4, participants in the *Fear of Conflict* condition were twice as likely to choose the non-zero-sum negotiation (67%, n = 56) over the zero-sum negotiation (33%, n = 28, *χ*^*2*^(1, 83) = 9.33, *p* = 0.002). In contrast, participants in the *No Fear* condition were equally likely to choose the non-zero-sum negotiation (48%, n = 38) as the zero-sum one (52%, n = 41, *χ*^*2*^(1, 78) = 0.11, *p* = 0.736). Thus, whereas participants were averse to zero-sum situations when they worried about conflict, they did not exhibit such aversion when prompted to disregard such fear.

### Study 6

Study 6 (*N* = 305) examines an important consequence of zero-sum aversion. Since people avoid zero-sum situations, organizations that compensate employees in such a manner (e.g., by evaluating employees relative to each other and only rewarding a set number of them) may need to offer much higher salaries to remain competitive and curb attrition. Indeed, although participants in Study 3A exhibited substantial zero-sum aversion, their aversion gradually weakened as the expected value of the zero-sum lottery increased. Similarly, companies like Amazon and Microsoft (which often conduct relative evaluations) and prestigious law firms (which famously evaluate junior associates relative to each other), may need to offer higher compensation to offset the perceived cost of interpersonal conflict.

To examine this, we asked a cohort of MBA students who were applying for internships to read a job description and report the base compensation (i.e., salary) that they would require. Participants were randomly assigned to one of three conditions. In the *zero-sum condition*, they read that the company evaluates employees “based on their relative performance” (which determines, among other things, their annual bonuses, above and beyond their base salary) so that they always know where they stand relative to their peers. In the *non-zero-sum condition*, they read that the company’s evaluations are based on “absolute performance” so that employees know where they stand “relative to these organizational milestones.” In the *control condition,* participants were not given any information about the evaluation process.

**Figure 5:**
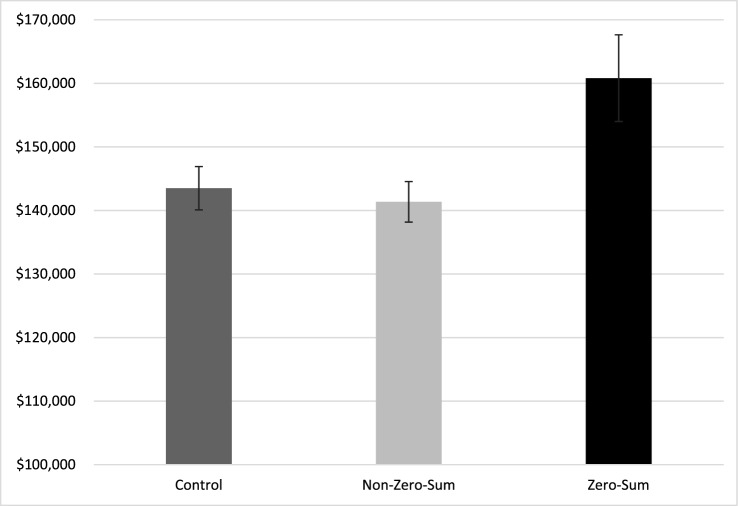
The base wage (i.e., salary) requirements of MBA students to work for the organization in each condition (Study 6). Errors bars represent standard errors (SEM).

We predicted that participants would demand a higher base salary to work at a company where the process of resource allocation is zero-sum. Indeed, a one-way ANOVA revealed a significant effect of condition on wage requirements, *F*(2, 302) = 4.95, *p* = 0.008 (Fig. 5). On average, participants demanded ~14% higher wages in the *zero-sum condition* (M = $160,817, SD = $69,547) than the *non-zero-sum condition* (M = $141,369, SD = $32,342), *p* = 0.013, and the *control condition* (M = $143,500, SD = $33,713), *p* = 0.036, which did not differ from each other, *p* > 0.999. Notably, this effect was exhibited even when controlling for participants’ beliefs regarding how well they will perform relative to their peers, including their self-esteem and their tendency to view themselves as smarter, kinder, and more skillful and attractive than their peers (*F*(2,299) = 5.68, *p* = 0.004). Regardless of their beliefs about how their performance would compare to their peers’ performance, participants demanded much higher wages to work in an environment where resources are allocated in a zero-sum manner.

## General discussion

Across different contexts (negotiations, economic games, market entry decisions, performance reviews, job applications), samples (online samples, a community sample, professional MBA students), and research designs (within- and between-participants, including real and hypothetical decisions), we found consistent evidence for zero-sum aversion—the avoidance of situations that are (or are believed to be) zero-sum. Notably, this was exhibited beyond any individual difference in social preferences, highlighting the role of situational factors in shaping people’s aversion to zero-sum resource allocations. Finally, both when doing so meant lower expected value and when making decisions for other people, concerns about conflict increased people’s zero-sum aversion.

A series of supplemental studies reported in the SOM further examine the robustness of this effect and rule-out several alternative explanations. We find that zero-sum aversion is not due to avoiding responsibility over others’ outcomes (Supplemental Studies S3A-S3C), a fear of appearing selfish or unfair (Supplemental Study S3D), an aversion to interdependence (Supplemental Study S3E) or inequity (Supplemental Study S3F), or participants’ affective expectations (Supplemental Studies S3G and S3H). Independent of the *outcome* of the allocation of a given resource, we find that people are averse to the *process* of zero-sum resource allocation.

Importantly, zero-sum aversion is both theoretically and empirically distinct from competition/tournament avoidance. First, we found that zero-sum aversion occurs whenever people’s outcomes are inversely related, regardless of whether or not these outcomes are determined through competition. For instance, participants exhibited zero-sum aversion even when deciding between different types of negotiations or, more tellingly, when deciding between different games of chance (i.e., where outcomes are completely determined by luck). Thus, while the two may be related, the evidence suggests that zero-sum aversion is a broader phenomenon than mere competition avoidance. Second, although a vast literature has documented evidence of socially structured gender differences in competition avoidance^25–29^, our studies suggest that zero-sum aversion is not moderated by gender (Table S4 in the SOM). Finally, whereas competition avoidance narrowly applies to choices about one’s own outcomes, we find that zero-sum aversion is exhibited *even when people make decisions for other people* (Study 4C), suggesting a much broader phenomenon.

Relatedly, our findings illustrate why people *do* sometimes willingly enter zero-sum situations, such as when they choose to engage in competitive (and often zero-sum) sports. As noted, we find that zero-sum aversion is at least partially due to people’s fear of conflict and that reducing such concerns about conflict increases their willingness to enter zero-sum situations. Accordingly, it is noteworthy that almost every type of competitive sport has strict rules and regulations that explicitly discourage conflict, prohibit violence and intimidation, and sanction undue aggression. Thus, zero-sum aversion is less likely to occur in organized sports exactly because such events eliminate (or subsntatially reduce) concerns about conflict. Consequently, eliminating people’s concerns about conflict in any zero-sum situation may reduce their aversion to it.

## Implications and future directions

Our findings are important for understanding zero-sum dynamics in interpersonal and intergroup conflict. As shown, in addition to avoiding *objectively* zero-sum situations, people also avoid situations they *subjectively* view as zero-sum. Since merely viewing situations as zero-sum fosters avoidance, exploring when and why people exhibit zero-sum beliefs and orienting them towards the possibility of non-zero-sum outcomes may reduce such aversion. Thus, our work extends research on zero-sum beliefs^5,11,30,31^ by highlighting the need to examine *whether* people engage in zero-sum relationships in the first place.

This paper is the first to document zero-sum aversion, and future research could benefit from examining potential moderators of this phenomenon. For instance, although zero-sum aversion was not moderated by gender and only weakly moderated by social preferences, future research could explore the effect of cultural^32–35^ and individual^36^ differences in people’s approach to conflict. Nevertheless, our findings make clear that beyond individual differences, fully understanding people’s preferences requires considering a situation’s characteristics (i.e., whether it is, or believed to be, zero-sum).

Furthermore, by understanding when and why people avoid zero-sum situations, our findings help explain why such situations tend to be rife with violence and animosity. Since people fear that zero-sum situations will involve conflict, they may preemptively approach them with heightened aggression, distrust, and hostility^5,37–39^.

Second, by impeding people’s willingness to negotiate, zero-sum aversion may lead people to overlook potential opportunities for both material and symbolic gains. As shown, merely seeing social relations as zero-sum inhibits people from initiating negotiations. Yet, because most negotiations combine both zero-sum and non-zero-sum elements, such aversion may lead people to refrain from negotiating even when they can benefit from doing so.

Third, by basing decisions on expectations of conflict rather than value, zero-sum aversion may promote ineffective resource allocations. For instance, people may forego lucrative opportunities that inversely link their and others’ outcomes, “leaving money on the table” by not approaching the table in the first place. At the same time, although they might do so reluctantly, people may still be willing to engage in zero-sum situations when these offer sufficiently high payoffs. Consequently, groups and organizations that reward people in zero-sum ways bear a substantial cost to attract new members. In contrast, by rewarding people in non-zero-sum ways, organizations may be able to offer environments that are (or are perceived to be) less riddled with conflict.

Of course, people may experience positive utility from avoiding conflict, and zero-sum aversion may reflect an attempt at maximize one's overall utility even at the expense of narrower, economic utility. Accordingly, zero-sum aversion may be considered ‘irrational’ only in very rare situations in which conflict is materially inconsequential, such as in anonymous, one-shot economic games that are stripped of any reputational concerns. Thus, rather than making normative claims about whether people *ought to* avoid zero-sum situations, our findings offer a descriptive claim about when (and why) people do so. Clearly, whether people view a situation as a zero-sum game determines whether they want to play in the first place.

## Materials and methods

For all studies, we report all measures and conditions. Sample sizes were determined in advance and analyses were conducted after data collection was complete. We preregistered Studies 2C, 3A, 3B, 4B, and 4C (see SOM). Materials and data are available at the Open Science Framework: https://osf.io/74bnw/?view_only=d3c2be0123db4d22887fa03aa27f09dc.

All studies were carried out in accordance with the relevant ethical guidelines and regulations for research with human subjects, were approved by the Institutional Reviews Board at Columbia University Human Research Protection Office (Protocol number: IRB-AAAS6914), and participants gave their informed consent prior to participation.

### Study 1A

#### Participants

Two hundred five MBA students completed the study (*M*_*age*_ = 28.31; 94 females, 110 males, 1 prefer not to answer; 52% White/European American, 3% Black/African American, 12% Hispanic/Latin American, 34% Asian/Asian American/Pacific, 4% Other), giving us 80% power to detect correlations as small as r = 0.19.

#### Materials and procedure

As part of a course survey, participants completed the Belief in Life as Zero-Sum Game Scale^5^, the Propensity to Initiate Negotiations Scale^20^, and a brief measure of the Big-Five personality traits^40^. See SOM for all items and reliability measures (Table S3).

### Study 1B

#### Participants

Two hundred two U.S. residents were recruited from Amazon’s Mechanical Turk (*M*_*age*_ = 39.34; 86 females, 111 males; 75.7% White, 10.2% Black, 6.8% Hispanic, 4.9% East Asian, 1.5% South Asian, < 1% Middle Eastern/Arabic), giving us 80% power to detect small effects (*dz* = 0.20) in a matched-pairs design.

#### Materials and procedure

Participants read about a company undergoing restructuring negotiations involving two main issues: “resource allocation” and “workflow integration.”

In the resource allocation negotiation, participants were told that “the company needs to determine how to allocate resources between departments,” that “resources are limited,” and that “the more one department receives, the less other departments can get.” Consequently, because the negotiation involved the distribution of resources, their responsibility would be to “advocate for as many resources as possible for your department” and “make sure that other departments don’t take resources away from you” (i.e., zero-sum).

In the workflow integration negotiation, participants were told that “the company needs to determine how to integrate the workflow between departments,” that “the potential for integration is limitless,” and that “effective integration can benefit all involved departments. Consequently, because the negotiation involved the integration of interests, their responsibility would be to “advocate for creating as much value as possible” and “make sure that other departments also benefit from the integration” (i.e., non-zero-sum).

Participants reported their willingness to represent their department at each negotiation (“To what extent would you like to represent your department at the discussions about resource allocation/workflow integration?” 1 = Not at all, 7 = Very much so)*.* In addition, to examine the effect of individual differences in social preferences on zero-sum aversion, participants completed the nine-item Social Value Orientation measure^30^, which we categorized as prosocial (n = 127) or individualistic (n = 64) based on the majority of their decisions. Only seven participants chose a mostly competitive orientation, thus barring statistical inferences about their preferences.

### Study 2A

#### Participants

One hundred U.S. residents were recruited from Amazon’s Mechanical Turk (*M*_*age*_ = 38.74; 46 females, 54 males; 82.5% White, 5.8% Black, 3.9% Hispanic, 4.9% East Asian, 1.9% South Asian, 1% Middle Eastern/Arabic), giving us 80% power to detect small effects (*dz* = 0.28) in a matched-pairs design.

#### Materials and procedure

Participants imagined choosing a location for a new business (a restaurant) between two potential options that vary on several dimensions: number of expected daily customers (i.e., likelihood of success), number of restaurants in town (i.e., the competition), and whether the market could be expanded (i.e., whether success is zero-sum).

In the non-zero-sum location, participants expected fewer daily customers, who would mainly come from expanding the market. They read that this location had six existing restaurants (with two more expected to open) and that they should expect “between 250 and 565 daily customers,” most of whom don’t typically frequent other restaurants. Consequently, because their outcomes would be independent from other restaurants’ outcomes, their business strategy would need to attract new customers rather than enticing clients from other businesses (i.e., non-zero-sum).

In the zero-sum location, participants expected more customers, but these would mainly come from other restaurants. They read that this location has fewer restaurants (three existing and one expected to open) and that they should expect substantially more business (“between 420 and 735 daily customers”), which would mostly come from people who already frequent other restaurants. Consequently, because their outcomes would be dependent on (and inversely linked to) other restaurants’ outcomes, their strategy would need to attract business away from them. Thus, despite having higher chances of success in this second location, their success would come at others’ expense (i.e., zero-sum). Participants reported how much they would want to open their restaurant at each location (“To what extent would you want to open your restaurant in Location A / Location B?” 1 = Not at all, 7 = Very much so)*.*

### Study 2B

#### Participants

One hundred five U.S. residents were recruited from Amazon’s Mechanical Turk (*M*_*age*_ = 39.67; 46 females, 55 males, 4 other/did not specify; 79.6% White, 4.6% Black, 6.5% Hispanic, 6.5% East Asian, 1.9% Indigenous/Native American), giving us 80% power to detect small effects (*dz* = 0.28) in a matched-pairs design and ratios as small as 1.46 in a one-sample chi-square design.

#### Materials and procedure

Participants read about a company that evaluates performance using two procedures, were explicitly told that each procedure is equally likely to result in favorable reviews, and indicated by which procedure they would prefer to be evaluated.

In the zero-sum procedure, the company inversely links employee outcomes by evaluating them relative to each other. Since the procedure rewards the highest ranked employees (and penalizes the lowest ranked employees), only a set number of rewards can be granted (i.e., zero-sum). In the non-zero-sum procedure, the company separates employee outcomes by independently evaluating each employee. Consequently, this procedure rewards all employees who outdo a given criteria and the number of rewards to be granted is not pre-determined or fixed (i.e., non-zero-sum). Participants reported how much they would want to be evaluated by each procedure (To what extent would you want your performance to be evaluated by the [“Ranked Success System” vs. “Rated Success System”]? 1 = Not at all, 7 = Very much so) and, indicated (in a forced-choice) their preference between them (I would choose the Ranked Success System vs. I would choose the Rated Success System based). They then explained their answers in an open-ended response.

### Study 2C

#### Participants

One-hundred ninety-nine participants were recruited through the community Behavioral Research Lab at Columbia Business School (*M*_*age*_ = 24.8; 124 females, 74 males, 1 other/did not specify; 37.2% White, 8% Black, 7% Hispanic, 34.2% East Asian, 17.1% South Asian, 1.5% Middle Eastern/Arabic, 8% Other), giving us 80% power to detect ratios as small as 1.32 in in a one-sample chi-square design.

#### Materials and procedure

Participants acted as interns for a magazine in a workplace simulation and were randomly assigned to one of two experimental conditions. In the *Difficult Task* condition, participants were instructed to count the number of characters and number of spaces in a series of long and grammatically complex sentences. In the *Simple Task* condition, they were instructed to simply count the number of words in a series of short sentences. After completing a trial run, participants evaluated their likelihood of outdoing others in this task: “How easy or difficult is this task?” (1 = Extremely easy; 5 = Extremely difficult), “In your opinion, how fast would you complete this task relative to other interns doing the exact same task?” (1 = Much slower than other interns; 5 = Much faster than other interns), and “In your opinion, how accurately would you complete this task relative to other interns doing the exact same task?” (1 = Much less accurate than other interns; 5 = Much more accurate than other interns). As intended, participants viewed the task as harder, and believed their performance would be worse than others’ performance, in the *Difficult Task* condition (M = 3.14, SD = 0.72) than the *Simple Task* condition (M = 2.35, SD = 0.52), *t*(197) = 8.97, *p* < 001, *d* = 1.27.

Following, participants completed the main dependent variable. They were told that, in the future, they may be invited to participate in an in-person follow-up study, in which they will complete similar tasks in dyads. They then chose in which session they would like to participate: a *zero-sum* session (i.e., relative speed and accuracy will determine participants’ bonuses, given to only one person in each dyad) or a *non-zero-sum* session (i.e., absolute speed and accuracy will determine participants’ bonuses, given to anyone who outperforms a set benchmark). Importantly, participants were informed that the chances of gaining a bonus would be identical in both sessions and that, after receiving their rewards, each pair will discuss their experiences. Participants then indicated their preference between the two future experimental sessions.

### Study 3A

#### Participants

Two hundred seven U.S. residents were recruited from Amazon’s Mechanical Turk (*M*_*age*_ = 40.54; 98 females, 102 males, 7 other/did not specify; 73.9% White, 9% Black, 4.7% Hispanic, 6.6% East Asian, 2.8% South Asian, 1% Indigenous/Native American, 1.9% Other), giving us 80% power to detect ratios as small as 1.31 in a chi-square design.

#### Materials and procedure

Participants completed the task as described in-text, making a series of decisions between two lotteries for either $1 or $5: a non-zero-sum lottery (where their chances of winning were independent from another player’s chances of winning) and a zero-sum lottery (where their chances of winning were inversely related to another player’s chances winning). In every decision, participants who chose the zero-sum option ended the experiment. Those who chose the non-zero-sum option saw two additional lotteries: the same *non-zero-sum* option as before and a new *zero-sum* option with better odds of winning. Participants completed these decisions five times, each with increasing odds of winning the zero-sum lottery. At the end of the experiment, we randomly entered one participant in a lottery based on their choice.

### Study 3B

#### Participants

Five hundred six U.S. residents were recruited from Amazon’s Mechanical Turk (*M*_*age*_ = 40.03; 265 females, 237 males, 4 other/did not specify; 73.8% White, 8% Black, 5.6% Hispanic, 7.1% East Asian, 2.4% South Asian, 0.4% Middle Eastern/Arabic, 1.5% Indigenous/Native American, 1.1% Other), giving us 80% power to detect ratios as small as 1.45–1.53 in each of five conditions in a one-sample chi-square design.

#### Materials and procedure

Participants completed the task as described in-text, making a single decision between two lotteries: a non-zero-sum lottery (where their chances of winning were independent from another player’s chances of winning) and a zero-sum lottery (where their chances of winning were inversely related to another player’s chances winning). Participants were randomly assigned to one of five conditions, in which we varied their odds of winning the *zero-sum* lottery. Whereas the non-zero-sum lottery always gave participants 50% chance of winning $1 (and this likelihood was *independent from* the other player’s likelihood of winning or losing), the zero-sum lottery offered participants 50%, 60%, 70%, 80%, or 90% of winning (and their likelihood was *inversely related to* the other player’s likelihood of winning or losing). Finally, we randomly entered three participants in lotteries based on their choice.

### Study 4A

#### Participants

One hundred one U.S. residents were recruited from Amazon’s Mechanical Turk (*M*_*age*_ = 40.74; 45 females, 56 males; 70.3% White, 5.9% Black, 5.9% Hispanic, 13.9% Asian, 1% American Indian/Alaskan Native, 1% Native Hawaiian/Pacific Islander American, 2% Other), giving us 80% power to detect medium effects (d = 0.56) in an independent sample t-test.

#### Materials and procedure

Participants were randomly assigned to read about one of two performance reviews from Study 2B. In the *zero-sum condition*, they read about a procedure that reviews employees relative to each other, inversely linking their outcomes and rewarding only a set number of high-ranked employees. In the *non-zero-sum condition*, they read about a procedure that independently reviews employees and rewards those who outdo absolute criteria, but in which there is no guarantee that anyone would do so.

Participants indicated how much they believed the procedure would create conflict using five-items: “harmful conflict,” “friction,” and “animosity” between employees at the company, “pit employees” against each other, and **“**put tension” on their relationships (1 = Not at all; 7 = Very much so; *α* = 0.98). Following, they reported how much they would want to be evaluated by the procedure (“To what extent would you like to be evaluated by the system that the company uses?” 1 = Not at all; 7 = Very much so).

### Study 4B

#### Participants

Two hundred fifty-one U.S. residents were recruited from Amazon’s Mechanical Turk (*M*_*age*_ = 40.15; 131 females, 118 males, 2 non-binary; 75.1% White, 6.1% Black, 5.8% Hispanic, 9.6% East Asian, 2.7% South Asian, < 1% Middle Eastern/Arabic, < 1% Other), giving us 80% power to detect medium effects (d = 0.36) in an independent sample t-test.

#### Materials and procedure

Participants were randomly assigned to read about one of two negotiations from Study 1B. In the *zero-sum condition*, they read that “the main issue to be discussed is resource allocation” and that representatives will need to “advocate for as many resources as possible” and “make sure that other departments don’t take resources away from [them].” In the *non-zero-sum condition,* participants read that “the main issue to be discussed is work integration” and that representatives will need to “advocate for integration that benefits everyone” and “make sure that [they] create as much value as possible.” Participants were warned in both conditions that the negotiations can become “heated” and that representing one’s division “is not going to be easy.” Following, participants indicated how much conflict the negotiations would create using the items from Study 4A **(**α = 0.94) and indicated their willingness to represent their department in them (“To what extent would you like to represent your department at these discussions?” 1 = Not at all; 7 = Very much so).

### Study 4C

#### Participants

Two hundred fifty-one U.S. residents were recruited from Amazon’s Mechanical Turk (*M*_*age*_ = 37.86; 105 females, 144 males, 2 prefer not to say; 72.5% White, 9.3% Black, 5.6% Hispanic, 7.1 East Asian, 3.0% South Asian, 1.1% American Indian/Alaskan Native, 1 < % Native Hawaiian/Pacific Islander American, < 1% Middle-Eastern/Arab, < 1% Other), giving us 80% power to detect medium effects (d = 0.36) in an independent sample t-test.

#### Materials and procedure

Participants were randomly assigned to read about one of two performance reviews from Study 2B. Unlike before, participants imagined they were managers at a company who were looking for new ways to evaluate their employees and were asked to consider the review process as a way of evaluating *other people* (i.e., their supervisees)*.* In the *zero-sum condition*, participants read about a procedure that reviews employees relative to each other, inversely linking their outcomes and rewarding only a set number of high-ranked employees. In the *non-zero-sum condition*, participants read about a procedure that independently reviews employees and rewards those who outdo absolute criteria.

Participants indicated how much they believe the procedure would create conflict among their employees using five-items: “harmful conflict,” “friction,” and “animosity” between employees at the company, “pit employees” against each other, and **“**put tension” on their relationships (1 = Not at all; 7 = Very much so; *α* = 0.98). Following, they reported whether they would choose to implement the review system for their employees (“How likely would you be to choose this system for your company?” 1 = Not at all likely; 7 = Very likely).

### Study 5A

#### Participants

Two hundred two U.S. residents were recruited from Amazon’s Mechanical Turk (*M*_*age*_ = 41.36; 123 females, 78 males; 80.4% White, 7.2% Black, 5.3% Hispanic, 3.8% East Asian, 1.4% South Asian, 1% Indigenous/Native American, < 1% Middle Eastern/Arabic, < 1% Other), giving us 80% power to detect small effects (*dz* = 0.20) in a matched-pairs design.

#### Materials and procedure

Participants read the material from Study 1B about negotiations concerning two issues—“resource allocation” (zero-sum) and “workflow integration” (non-zero-sum)—and were randomly assigned to one of two conditions. In the *Fear of Conflict* condition, participants read that their “company is a very friendly and collegial place to work” and that “whichever discussion you end up joining, your goal should focus on avoiding conflict.” They further read that “helping your department is obviously important, but it's not important enough to ruin your personal relationships in the organization” and that they “have been really successful at avoiding conflict, and you would like to keep it that way.” In the *No Fear* condition, participants read that their “company is a very goal-oriented environment” and that “whichever discussion you end up joining, your goal should focus on helping your department.” They further read that “your personal relationships in the organization are obviously important, but they are not important enough to jeopardize your devotion to your department” and that they “have been a really successful contributor to your department, and you would like to keep it that way, even at the cost of getting in conflict with others in the organization.” Following, participants reported their willingness to represent their department at each negotiation (“To what extent would you like to represent your department at the discussions about resource allocation/workflow integration?” 1 = Not at all, 7 = Very much so) and completed, as a manipulation check, the same measure of perceived conflict Studies 4A and 4B (Indeed, participants were more worried about the emergence of conflict when they believed that the company has a goal-oriented environment where people value performance over harming interpersonal relationships (M_No-fear-condition_ = 4.38, SD = 1.28) than when they believed that the company is a very collegial place where people shy from getting into conflict (M_Fear-of-conflict-condition_ = 3.95, SD = 1.25), *t*(199) = 2.43, *p* = 0.016, *d* = 0.34).

### Study 5B

#### Participants

Two hundred three U.S. residents were recruited from Amazon’s Mechanical Turk (*M*_*age*_ = 41.81; 107 females, 91 males, 4 Prefer not to say; 76.1% White, 6.6% Black, 7.5% Hispanic, 7.0% East Asian, < 1% South Asian, < 1% Indigenous/Native American, < 1% Middle Eastern/Arabic, < 1% Other), giving us 80% power to detect ratios as small as 1.32 in a chi-square design.

#### Materials and procedure

Participants read the same materials from Study 5A about negotiations involving two issues—“resource allocation” (zero-sum) and “workflow integration” (non-zero-sum)—and were randomly assigned to one of two conditions. In the *Fear of Conflict* condition, we made the participants’ concerns about conflict salient by asking them to imagine “that you were extremely concerned about whether these discussions could lead to conflict and about how they would impact your relationship with your colleagues.” In the *No Fear* condition, we deemphasized the importannce of conflict by alleviating participants’ concerns about it and asking them to imagine “that you didn't care at all about whether these discussions could lead to conflict or how they would impact your relationship with your colleagues.”

Following, participants chose between the two different negotiations in a trinary decision (“Which discussion would you choose to join?” The discussion about resource allocation, The discussion about workflow integration, or No Preference).

### Study 6

#### Participants

Three hundred five MBA students completed the study (*M*_*age*_ = 27.86; 117 females, 187 males, 1 prefer not to answer; 40% White/European American, 7% Black/African American, 11% Hispanic/Latin American, 33% Asian/Asian American/Pacific, 2% Middle Easter/Arab, 6% Other), giving us 80% power to detect small effects (f = 0.18) in a one-way omnibus ANOVA.

#### Materials and procedure

As part of a broader survey, participants read an excerpt from an ostensible job posting for Orion-Toren Consultants (OTC)—a “mid-sized, East coast-based boutique consulting company targeting recent MBA graduates”—and were randomly assigned to one of three conditions. In the zero-sum condition, participants read:

*At Orion-Toren, we believe that only the best consultants can make it in the long run. As a consultant at OTC, you will undergo our proprietary professional-development program and will be evaluated based on how you perform relative to your incoming group of 8-10 other consultants. Consultants are ranked relative to each other, and receive constant guidance and evaluation, so that you will always know where you stand relative to your immediate peers. Needless to say, we expect all of our consultants to strive to beat their relative peer group. We take these performance evaluations seriously and use them to determine our consultants' compensation packages. Consultants who outdo their immediate peers are handsomely rewarded on a semi-annual basis.*

In the non-zero-sum condition, participants read:

*At Orion-Toren, we believe that only the best consultants can make it in the long run. As a consultant at OTC, you will undergo our proprietary professional-development program and will be evaluated based on how you perform relative to predetermined set benchmarks. Consultants are evaluated relative to these absolute benchmarks, and receive constant guidance and evaluation, so that you will always know where you stand relative to these organizational milestones. Needless to say, we expect all of our consultants to strive to beat their benchmarks. We take these performance evaluations seriously and use them to determine tour consultants' compensation packages. Consultants who outdo their absolute benchmarks are handsomely rewarded on a semi-annual basis.*

Finally, in the control condition participants read:

*At Orion-Toren, we believe that only the best consultants can make it in the long run. As a consultant at OTC, you will undergo our proprietary professional-development program and will be evaluated based on how you perform. Consultants receive constant guidance and evaluation, so that you will always know where you stand. We take these performance evaluations seriously and use them to determine tour consultants' compensation packages. Consultants who perform well are handsomely rewarded on a semi-annual basis.*

After reading the job posting, participants were told that they learned through an acquaintance that the starting salary for consultant was, in the past, $115,000 and indicated how much money they would ask for to work at this company (“What is the minimum salary you would be willing to accept to take this job?”). In addition, participants completed a single-item measures of self-esteem (“Please rate how much you agree with the following statement: I have high self esteem” 1 = strongly disagree, 7 = strongly agree) and an eight-item measure of their self-perceptions as better-than-average (“For the following questions, please rate yourself in comparison to the other students in this class: Decision-Making, Bargaining, Intelligence, Driving, Health, Charity, Physical Attractiveness, Good Friends”).

## Supplementary Information

Supplementary Information.

## Data Availability

All data and materials are available at the Open Science Framework: https://osf.io/74bnw/?view_only=d3c2be0123db4d22887fa03aa27f09dc.
