# Supplementary material for: The fear of conflict leads people to systematically avoid potentially valuable zero-sum situations
Source: Sci Rep. 2022 Oct 26;12:17944. doi: 10.1038/s41598-022-22849-y (PMC9605973; doi:10.1038/s41598-022-22849-y)
Supplement: Supplementary file 1 — Supplementary Information. [file 41598_2022_22849_MOESM1_ESM.docx]

**Supplementary Materials**

1. Table S1. Regression results predicting concern about the social consequences of negotiations from participants’ zero-sum beliefs and their self-reported Big-Five personality traits (Study 1A)
2. Table S2. Regression results predicting concern about the social consequences of negotiations from participants’ zero-sum beliefs and their self-reported personality traits (Study 1A)
3. Table S3. Scale reliability for measures used in Study 1A.
4. Table S4. Exploring Moderation by Gender
5. **Supplemental** **Study S1**
6. **Supplemental** **Study S2**
7. **Supplemental** **Studies S3A-S3H:** Ruling out alternative explanations
   1. **Supplemental** **Study S3A**: Ruling out avoidance of responsibility over others’ outcomes
   2. **Supplemental** **Study S3B**: Further ruling out avoidance of responsibility over others’ outcomes
   3. **Supplemental** **Study S3D**: Ruling out fear of appearing selfish/unfair
   4. **Supplemental** **Study S3E**: Ruling out reluctance to interdependence
   5. **Supplemental** **Study S3F**: Ruling out inequity aversion
   6. **Supplemental** **Study S3G**: Ruling out affective expectations
   7. **Supplemental** **Study S3H**: Further ruling out affective expectations
8. **Supplemental** **Study S4**: Expectation of conflict in an economic game
9. Figure S1*.* The mediating role of perceived conflict on the relationship between type of negotiation (zero-sum vs. non-zero-sum) and zero-sum aversion (Study 4B).
10. Figure S2*.* The mediating role of perceived conflict on the relationship between type of review process (zero-sum vs. non-zero-sum) and zero-sum aversion (Study 4C).
11. Figure S3. Research design of Study S1. At each stage, participants who chose the non-zero-sum option (two separate coin-flips, left side) continued to the next choice, where the expected value of the zero-sum option increased in $1 increments. Participants who chose the zero-sum option (one coin-flip, right side) ended the study
12. Figure S4. The cumulative likelihood of choosing the zero-sum option at each incremental $1 payoff increase of the zero-sum option (Study S1).
13. Figure S5. The cumulative likelihood of choosing the zero-sum option at each incremental 10% likelihood increase of the zero-sum option (Study S2)
14. Figure S6. The non-zero-sum (left) and zero-sum (right) options in Studies S3A (top), S3E (middle), and S3F (bottom).
15. Pre-registration links

**Table S1. Regression results predicting concern about the social consequences of negotiations from participants’ zero-sum beliefs and their self-reported Big-Five personality traits (Study 1A)**

|  | **Model 1** | | | **Model 2** | | |
| --- | --- | --- | --- | --- | --- | --- |
|  | ***b*** | **se** | ***p*** | ***b*** | **se** | ***p*** |
| **Constant** | 3.181 | 0.21 | < .001 | 4.470 | 0.68 | < .001 |
| **Zero-Sum Beliefs** | 0.282 | 0.07 | < .001 | 0.198 | 0.07 | 0.005 |
| **Openness** |  |  |  | -0.172 | 0.07 | 0.019 |
| **Neuroticism** |  |  |  | 0.124 | 0.05 | 0.023 |
| **Agreeableness** |  |  |  | 0.175 | 0.07 | 0.010 |
| **Extraversion** |  |  |  | -0.186 | 0.05 | < .001 |
| **Conscientiousness** |  |  |  | -0.099 | 0.07 | 0.129 |

*Table S1.* Regression Model 1 predicts participant’s beliefs that negotiations have social consequences related to conflict using zero-sum beliefs as a predictor. In Model 2, we add participant’s self-report Big-Five personality traits as predictors. In both models, zero-sum beliefs predict the extent people anticipate negotiations will create interpersonal conflict.

**Table S2. Regression results predicting concern about the social consequences of negotiations from participants’ zero-sum beliefs and their self-reported personality traits (Study 1A)**

|  | *b* | *se* | *p* |
| --- | --- | --- | --- |
| Constant | 2.44 | 1.00 | 0.016 |
| **Zero-Sum Beliefs** | **0.20** | **0.07** | **0.005** |
| Conventional | 0.10 | 0.05 | 0.043 |
| Open | -0.06 | 0.08 | 0.433 |
| Calm | 0.00 | 0.08 | 0.973 |
| Anxious | 0.10 | 0.06 | 0.086 |
| Critical | -0.03 | 0.05 | 0.489 |
| Warm | 0.18 | 0.07 | 0.006 |
| Reserved | 0.06 | 0.06 | 0.340 |
| Extraverted | -0.13 | 0.07 | 0.050 |
| Dependable | -0.01 | 0.07 | 0.856 |
| Disorganized | 0.09 | 0.06 | 0.161 |

**Table S3. Scale reliability for measures used in Study 1A.**


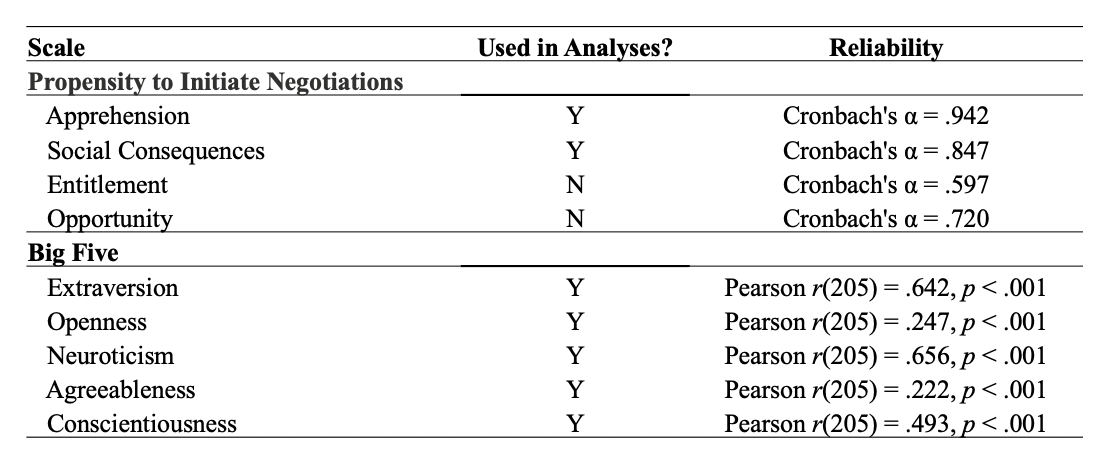


**Table S4. Exploring Moderation by Gender**

| **Study** | **Moderation by gender** | **Explanation** |
| --- | --- | --- |
| 1A | No | No interaction of zero-sum beliefs and gender on the apprehension to initiate negotiations (*b*=.13, *se* = .20, *p* = .523) |
| 1B | No | No interaction of negotiation type and gender on the aversion to the negotiation (*F*(1,98) = 1.10, *p* = .295) |
| 2A | No | No interaction of review type and gender on aversion to performance review (*F*(1,99) = 1.04, *p* = .319) |
| 2B | No | No interaction of market entry context and gender on aversion to enter market (*F*(1,98) = .07, *p* = .786) |
| 2C | Yes | Women exhibited significantly greater zero-sum aversion than men (χ= 12.16, p < .001). Across conditions, 16.9% of women chose the zero-sum option, while 39.2% of men chose the zero-sum option. |
| 3A | No | No gender differences in choosing the zero-sum lottery (*χ*= 1.42, *p* = .234) |
| 3B | Mixed findings | No gender differences in choosing the zero-sum lottery in the 50/50 condition (*χ*= 0.003, *p* = .953), 70/30 condition (*χ*= 0.17, *p* = .685), and 90/10 condition (*χ*= 0.03, *p* = .854). Women were more averse to the zero-sum lotteries in the 60/40 condition (*χ*= 4.45, *p* = .035) and 80/20 condition (*χ*= 8.34, *p* = .004). |
| 4A | No | No interaction of condition and gender on aversion to the evaluation (*F*(1,97) = 0.23, *p* = .637) |
| 4B | No | No interaction of condition and gender on aversion to negotiating (*F*(1,245) = 0.55, *p* = .459) |
| 4C | No | No interaction of condition and gender on aversion to negotiating (*F*(1,248) = 1.12, *p* = .291) |
| 5A | No | No interaction of negotiation type and gender on aversion to negotiating (*F*(2,198) = 0.197, *p* = .821) |
| 5B | No | No interaction of negotiation type and gender on aversion to negotiating (*χ*= 1.14, *p* = .287) |
| 6 | No | No interaction of condition and gender on wage requirements (*F*(2,298) = 0.48, *p* = .618) |
| S1 | No | No gender differences in choosing the zero-sum lottery (*χ*= 0.30, *p* = .585) |
| S2 | No | No gender differences in choosing the zero-sum lottery (*χ*= 3.06, *p* = .080) |
| S3A | Yes | Men exhibited greater zero-sum aversion than women (*χ*= 6.34, *p* = .012). While 4.7% of men chose the zero-sum lottery, 22.8% of women chose the zero-sum lottery. |
| S3B | No | No gender differences in choosing the zero-sum lottery (*χ*= 0.49, *p* = .485) |
| S3C | No | No gender differences in choosing the zero-sum lottery (*χ*= 1.00, *p* = .317) |
| S3D | Yes | Women exhibited greater zero-sum aversion than men (χ= 6.16, p = .013). While 17.6% of women chose the zero-sum lottery, 40.0% of men chose the zero-sum lottery. |
| S3E | No | No gender differences in choosing the zero-sum lottery (*χ*= 0.59, *p* = .443) |
| S3F | No | No gender differences in choosing the zero-sum lottery (*χ*= 0.004, *p* = .951) |
| S3G | No | No gender differences in choosing the zero-sum lottery (*χ*= 1.34, *p* = .512) |
| S3H | No | No gender differences in choosing the zero-sum lottery (*χ*= 0.04, *p* = .844) |
| S4 | No | No gender differences in choosing the zero-sum lottery (*χ*= 3.51, *p* = .173) |

**Supplemental** **Study S1**

We examine zero-sum aversion in a choice between two economic games: a zero-sum game that inversely links participants’ payoffs to another player’s payoffs and a non-zero-sum game that separates the players’ payoffs from each other. We predicted that participants would choose to separate their and others’ payoffs rather than inversely link them in a zero-sum game.

**Method**

**Participants**. One hundred ten U.S. residents were recruited from Amazon’s Mechanical Turk (*M_age_* = 35.78; 40 females, 58 males, 12 other/did not specify; 71.2% White, 6.7% Black, 7.7% Hispanic, 11.5% East Asian, 1% South Asian, 2% Other), giving us 80% power to detect ratios as small as 1.45 in a one-sample chi-square design.

**Materials and Procedure**. Participants indicated which of two games they would choose to play: a *non-zero-sum* game or a *zero-sum* game. In the *non-zero-sum* game, participants imagined that they and another player each flipped a coin, with 50% chance of gaining or losing $1. Since separate coin-flips guarantee that players’ outcomes are independent from each other, this game is non-zero-sum, leading to mixed gains (one player gains while the other loses), joint gains (both win $1), or joint losses (both lose $1). In the *zero-sum* game, participants imagined flipping one coin to determine both players’ outcomes, with 50% chance of gaining $1 while the other player loses $1 and 50% chance of losing $1 while the other player gains $1. Since this game inversely links the players’ outcomes, it is inherently zero-sum and does not offer opportunity for joint gains or losses

At this point, the experiment ended for participants who chose the zero-sum game. Participants who chose the non-zero-sum game were presented with two additional options: the same *non-zero-sum* game from before (independently giving each player 50% chance of gaining or losing $1) and a new *zero-sum* game with potentially higher payoff (giving them 50% chance of gaining $2 while the other player loses $2 and 50% chance of losing $1 while the other player gains $1). Although the zero-sum game still offset participants’ gains with the other player’s losses, it also gave them a higher expected value: a chance of gaining $2 at the risk of losing $1 (Figure S3).

We repeated this procedure three times. Each time, participants who chose the zero-sum game ended the experiment. Those who chose the lower value, non-zero-sum game faced two additional options: the same *non-zero-sum* game (giving each player 50% chance of gaining or losing $1) and a new *zero-sum* game with higher payoffs in $1 increments: a zero-sum game that gave participants 50% chance of gaining $3 (while the other player loses $3) and 50% chance of losing $1 (while the other player gains $1), a *zero-sum* game that gave them 50% chance of gaining $4 (while the other player loses $4) and 50% chance of losing $1 (while the other player gains $1) and a *zero-sum* game with 50% chance of gaining $5 (while the other player loses $5) and 50% chance of losing $1 (while the other player gains $1). The experiment ended after the 5^th^ round regardless of which game participants chose. Finally, participants reported their age, gender, and race/ethnicity.

**Results**

First, we examined participants’ choice in the first decision, when the two games were equivalent. Although both games at this point offered equal chance of winning or losing $1, participants exhibited zero-sum aversion—choosing the *non-zero-sum* game (67%, n = 74) significantly more often than the *zero-sum* game (33%, n = 37), *χ^2^*(1,110) = 12.57, *p* < .001.

Figure S4 presents participants’ choices at each subsequent payoff increase of the zero-sum game. Of the 74 participants who chose the non-zero-sum game in the first choice, 68% (n = 50) still opted for this game in the second choice rather than the zero-sum game that offered $0.50 more in expected value, *χ^2^*(1,73) = 9.33, *p* = .002. Of these, 69% (n = 34) still chose the non-zero-sum game in the third choice rather than the zero-sum game that offered $1 more in expected value, *χ^2^*(1,48) = 7.56, *p* = .006. Of these participants, 74% (n = 25) still chose the non-zero-sum game in the fourth choice rather than the zero-sum game that offered $1.50 more in expected value, *χ^2^*(1,33) = 7.84, *p* = .005. Finally, of the 25 participants who chose the non-zero-sum game in the first four choices, 76% (n = 19) still preferred this game in the fifth (and final) choice rather than the zero-sum game that offered $2 more in expected value, *χ^2^*(1,24) = 7.10, *p* = .008. Thus, although the proportion of participants choosing the zero-sum game gradually increased as its expected value rose relative to the non-zero-sum game, participants exhibited zero-sum aversion in anonymous, one-shot games even when this could cost them $0.50, $1, $1.50, or $2. On average, participants needed 62% more in expected value to switch from the non-zero-sum game to the zero-sum game (a conservative estimate, treating participants who chose the non-zero-sum game in the final choice as willing to switch to the zero-sum game if its expected value rose by even $0.01 more).

**Supplemental** **Study S2**

**Method**

**Participants**. Two hundred fourteen U.S. residents were recruited from Amazon’s Mechanical Turk (*M_age_* = 38.83; 76 females, 122 males, 15 other/did not specify; 78.1% White, 4.4% Black, 3.4% Hispanic, 10.7% East Asian, 2% South Asian, 0.5% Middle Easter/Arabic, 1% Other), giving us 80% power to detect ratios as small as 1.31 in a one-sample chi-square design.

**Materials and Procedure.** Participants made a series of decisions between two lotteries. They were told that three people will be randomly entered in a lottery based on their choice.

In each decision, participants chose between a *non-zero-sum* option and a *zero-sum* option. In the *non-zero-sum* option, each player would be entered into a separate lottery, with 50% chance of gaining or losing $1. This option guarantees that players’ payoffs are independent from each other and is therefore non-zero-sum, leading to mixed gains (one player gains while the other loses), joint gains (both players gain), or joint losses (both players lose). In the *zero-sum* option, both players would be entered into the same lottery, with 50% chance of gaining $1 while the other player loses $1 and 50% chance of losing $1 while the other player gains $1.

At this point, the experiment ended for participants who chose the zero-sum lottery. Participants who chose the non-zero-sum lottery saw two additional options: the same *non-zero-sum* lottery from before (independently giving each player 50% chance of gaining or losing $1) and a new *zero-sum* lottery with better odds of winning (giving participants 60% chance of gaining $1 while the other player loses $1 and 40% chance of losing $1 while the other player gains $1). Although this zero-sum lottery inversely links the players’ payoffs, it also offers participants better chances of winning than losing.

We repeated this procedure three times. Each time, participants who chose the safer, zero-sum option ended the experiment. Those who chose the riskier, non-zero-sum lottery faced two additional options: the same *non-zero-sum* lottery (giving each player 50% chance of gaining or losing $1) and a new *zero-sum* lottery with even better odds of winning: a *zero-sum* lottery that gave participants 70% chance of gaining $1 (while the other person loses $1) and 30% chance of losing $1 (while the other person gains $1), a *zero-sum* lottery that gave them 80% chance of gaining $1 (while the other person loses $1) and 20% chance of losing $1 (while the other person gains $1), and a *zero-sum* lottery that gave them 90% chance of gaining $1 (while the other person loses $1) and 10% chance of losing $1 (while the other person gains $1). The experiment ended after the 5^th^ round regardless of which lottery they chose, and participants indicated their age, gender, and race/ethnicity. Finally, we randomly chose three participants, paired them with another person, and entered them into the lottery of their choice.

**Results**

First, we examined the percentage of participants who, in the first decision, chose the non-zero-sum lottery over the zero-sum lottery. Although both lotteries at this stage offered equal chance of winning, and despite playing for real monetary outcomes, participants chose the *non-zero-sum* lottery (79%, n = 168) significantly more often than the *zero-sum* lottery (21%, n = 46), *χ^2^*(1,213) = 73.92, *p* < .001.

Figure S5 presents participants’ choice as the odds of winning the zero-sum lottery increased. Of the 168 participants who chose the non-zero-sum lottery in the first choice, 74% (n = 123) still opted for it in the second choice rather than the zero-sum option that offered 10% higher chances of winning, *χ^2^*(1,167) = 41.79, *p* < .001. Of these, 73% (n = 91) still chose the non-zero-sum lottery in the third choice rather than the zero-sum lottery that offered 20% higher chances of winning, *χ^2^*(1,122) = 26.98, *<* .001. Of these 91 participants, 69% (n = 63) still chose the non-zero-sum lottery in the fourth choice rather than the zero-sum lottery that offered 30% higher chances of winning, *χ^2^*(1,92) = 13.82, *p* < .001. Finally, of the 63 participants who chose the non-zero-sum lotteries in the previous four choices, 83% (n = 52) still preferred this lottery in the fifth (and final) choice over the zero-sum lottery that offered 40% higher chances of winning, *χ^2^*(1,51) = 28.98, *p* < .001. Thus, while the proportion of participants who chose the zero-sum lottery gradually increased with the odds of winning it, participants exhibited zero-sum aversion even when the chances of winning the zero-sum lottery were 10%, 20%, 30% and 40% higher. On average, participants needed 20.7% higher chances of winning to switch from the non-zero-sum to the zero-sum lottery (a conservative estimate, treating participants who chose the non-zero-sum lottery in their final choice as willing to switch to the zero-sum option if its chances of winning rose by an additional 0.1%.).

**Supplemental** **Studies S3A-S3H**

Studies S3A-S3H examine zero-sum aversion in a sequence of controlled experiments which rule-out potential alternative explanations. In a series of one-shot anonymous economic games, we examine whether participants prefer non-zero-sum situations even when they offer lower expected value. Moreover, we examine whether zero-sum aversion is due to a desire to not be responsible for others’ outcomes (Studies S3A-S3C), a fear of appearing selfish (Study S3D), an aversion to interdependence (Study S3E) or inequality (Study S3F), or a belief that winning and losing feel worse in zero-sum situations (Studies S3G and S3H). In all studies, we predicted that participants would avoid zero-sum situations even when it is costly to do so.

**Study S3A: Ruling out avoidance of responsibility over others’ outcomes**

Participants may prefer non-zero-sum games to zero-sum games because they want to give other people the agency to control their own outcomes. Specifically, because other people’s outcomes in zero-sum games are often determined by one’s own actions, people might simply wish to maximize others’ agency and therefore be averse to such situations. To examine this, participants in Study S3A chose between two games where each player was responsible for their own coin-flip, but where payoffs were either zero-sum or non-zero-sum.

**Method**

**Participants**. One hundred U.S. residents were recruited from Amazon’s Mechanical Turk (*M_age_* = 42.93; 57 females, 43 males; 84% White, 4% Black, 4% Hispanic, 6% East Asian, 1% South Asian, 1% Middle Eastern/Arabic), giving us 80% power to detect ratios as small as 1.48 in a one-sample chi-square design.

**Materials and Procedure**. Participants chose between two games: a *non-zero-sum* game and a *zero-sum* game. In the non-zero-sum game, each player would be responsible for flipping their own coin and payoffs would be separately determined by each player’s coin-flip, independently giving each player 50% chance of gaining or losing $1. In the zero-sum game, each player would still be responsible for flipping their own coin, but payoffs would depend on the outcomes of both coin-flips (Figure S6): If both players flipped the same outcome (Heads or Tails), the participant would gain $1 but the other player would lose $1. If the players flipped different outcomes (one flipped Heads while the other flipped Tails), the participant would lose $1, but the other play would win $1. This set-up gave participants 50% chance of gaining while the other player loses and 50% chance of losing while the other player gains. After making their choice, participants indicated their age, gender, and race/ethnicity.

**Results**

If participants are truly averse to having their outcomes inversely linked to another player’s outcomes, they should exhibit zero-sum aversion even though each player in the zero-sum game is responsible for flipping their own coin. Indeed, although both games offered the same likelihood of gaining or losing, and although each player flipped their own coin, participants overwhelmingly chose the non-zero-sum game (85%, n = 85) over the zero-sum game (15%, n = 15), *χ^2^*(1,99) = 54.09, *p* < .001. Thus, participants again exhibited zero-sum aversion, opting to separate their and others’ gains and losses.

**Study S3B: Further ruling out avoidance of responsibility over others’ outcomes**

To further examine whether zero-sum aversion is due to differences in who ‘control’s each game (i.e., who flips the coins), we told participants in Study S3B that they would be responsible for flipping both coins in the non-zero-sum game—one coin for themselves and a different coin for the other player. If participants don’t want to affect the other player’s outcomes, then having them flip both coins in the non-zero-sum game should reduce their preference for it. In contrast, if zero-sum aversion accounts for their choice, then participants should opt for the separate, non-zero-sum coin-flips rather than the combined, zero-sum coin-flip regardless of who is responsible for flipping the coins.

**Method**

**Participants**. One hundred fifteen U.S. residents were recruited from Amazon’s Mechanical Turk (*M_age_* = 39.04; 36 females, 64 males, 15 other/did not specify; 82.9% White, 5.7% Black, 2.9% Hispanic, 6.7% East Asian, 1% South Asian, 1% Indigenous/Native American). This sample gives us 80% power to detect ratios as small as 1.44 in a one-sample chi-square design.

**Materials and Procedure**. Participants indicated which of two games they would choose to play with another person: separate *non-zero-sum* coin-flips (independently giving each player 50% chance of gaining or losing $1) or a combined *zero-sum* coin-flip (giving them 50% chance of gaining $1 while the other player loses $1 and 50% chance of losing $1 while the other player gains $1). In contrast to Study S1, participants were told that in both games they would be responsible for flipping all the coins—the two coins in the non-zero-sum game and the one coin in the zero-sum game—so that their actions would determine the other player’s outcomes. After indicating which game they would like to play, participants explained their decision and indicated their age, gender, and race/ethnicity.

**Results**

If zero-sum aversion is due to participants’ reluctance to control the other player’s outcomes, then they should be indifferent between the two options in Study S3B, where they are responsible for flipping the coins regardless of which game they choose. In contrast, if participants are truly averse to having their outcomes inversely linked to another player’s outcomes, they should exhibit zero-sum aversion even when the non-zero-sum game requires that they flip a (separate) coin to determine the other player’s outcome. Indeed, despite having to affect the other player’s outcomes, participants were significantly more likely to choose the non-zero-sum, two separate coin-flips (61%, n = 70) than the zero-sum, one combined coin-flip (39%, n = 45), *χ^2^*(1,115) = 5.48, *p* = .019. Thus, participants again exhibited zero-sum aversion, opting to separate their gains and losses from others’ gains and losses.

**Study S3C: Further ruling out avoidance of responsibility over others’ outcomes**

We further examined whether zero-sum aversion is due to differences in who is responsible for the outcomes game (i.e., who flips the coins) by telling participants in Study S3C that *the other player* would be flipping the coin in the zero-sum game to determine both players’ payoffs. If participants don’t want to affect the other player’s outcomes, then having the other player flip the coin in the zero-sum game should reduce their aversion to it.

**Method**

**Participants**. One hundred twenty-three U.S. residents were recruited from Amazon’s Mechanical Turk (*M_age_* = 38.78; 49 females, 52 males, 22 did not specify; 79.4% White, 4.7% Black, 5.6% Hispanic, 7.5% East Asian, 1.9% South Asian, <1% Middle-Eastern/Arab). This sample gives us 80% power to detect ratios as small as 1.42 in a one-sample chi-square design.

**Materials and Procedure**. Participants indicated which of two games they would choose to play with another person: separate *non-zero-sum* coin-flips (independently giving each player 50% chance of gaining or losing $1) or a combined *zero-sum* coin-flip (giving them 50% chance of gaining $1 while the other player loses $1 and 50% chance of losing $1 while the other player gains $1). In contrast to Study S1, participants were told that the other player would be responsible for flipping the coin in the zero-sum game, so that the other player’s actions would determine both their own and the the other person’s outcomes. After indicating which game they would like to play, participants explained their decision and indicated their age, gender, and race/ethnicity.

**Results**

If zero-sum aversion is due to participants’ reluctance to control the other player’s outcomes, then they should be indifferent between the two options in Study S3C, where the other person is responsible for flipping the coins in the zero-sum game. In contrast, if participants are truly averse to having their outcomes inversely linked to another player’s outcomes, they should exhibit zero-sum aversion even when outcomes in the zero-sum game are determined by the other player’s action. Indeed, participants were significantly more likely to choose the non-zero-sum, two separate coin-flips (63.4%, n = 78) than the zero-sum, one combined coin-flip (36.6%, n = 45), *χ^2^*(1,122) = 8.85, *p* = .003. Thus, participants again exhibited zero-sum aversion, opting to separate their gains and losses from others’ gains and losses.

**Study S3D: Ruling out fear of appearing selfish/unfair**

People often behave in seemingly prosocial ways not because of some inherent generosity but because of their beliefs regarding how others s expect them to behave. For instance, when participants in a Dictator Game can pay to exit the game without the recipient’s knowledge, a sizable minority choose to do so, effectively paying money to not appear selfish (Dana, Cain, & Dawes, 2006; Cain, Dana, & Newman, 2014). Similarly, using a modified version of the ‘Ultimatum Game,’ Shalvi et al. (2011) found that people are willing to incur a cost to avoid appearing unfair, even when doing so ultimately harms another person.

In a similar vein, it is possible that people avoid zero-sum situations to not appear selfish. Even though both options offer them and the other person an equal chance of winning, participants may view the zero-sum option as *signaling* selfishness, especially when they are to ones responsible for deciding which game the two people will play. Thus, people may avoid zero-sum games because they worry that others might view them as selfish for choosing them. (The being said, such concerns about appearing selfish and unfair or about not wishing to be responsible for the other person’s outcomes are only applicable to the coin-flip studies, in which participants made a choice that would affect which game they and another person would play. This concern is not applicable to Studies 1A and 1B in the main manuscript, in which participants chose which workplace to join and where to open a new business, since their decisions in each context were independent from all other potential ‘players.’)

To examine this, participants in Study S3C were told that after making their decision, they will be matched with another participant who chose the same game as they did. Since this design guarantees that only people who willingly choose to play the zero-sum will do so, it should alleviate concerns about being or appearing selfish and unfair.

**Method**

**Participants**. One hundred two U.S. residents were recruited from Amazon’s Mechanical Turk (*M_age_* = 40.62; 51 females, 50 males, 1 other; 75.7% White, 6.5% Black, 5.6% Hispanic, 7.5% East Asian, 2.8% South Asian, <1% Middle Eastern/Arabic, <1% other), giving us 80% power to detect ratios as small as 1.48 in a one-sample chi-square design.

**Materials and Procedure**. Participants chose between a *non-zero-sum* game and a *zero-sum* game and were told that two participants would be randomly chosen at the end of the experiment and entered in the game of their choice. Participants were told that every participant was free to make their own choice and that, if picked, they will be matched with another participant who made the exact same choice as them. For example, if participants choose the zero-sum game and are randomly picked at the end of the experiment, they would be matched with another participant who also chose to play the zero-sum game.

Notice that this design alleviates any concerns about being perceived as selfish, unfair, or responsible for others’ outcomes. Specifically, since participants who choose the zero-sum game know that they would be paired with another person who voluntarily made the same choice as they did, they should not fear as being perceived as selfish or unfair by this person who also chose to play the zero-sum game. And, since each participant decides which game they would like to play, their choices only affect their own outcomes but not the other person’s outcome.

In the non-zero-sum game, each player’s payoffs would be separately determined by a different coin-flip, independently giving each player 50% chance of gaining or losing $1. In the zero-sum game, both players’ payoffs would be determined by the same coin flip, such that their outcomes would be inversely related, giving each player a 50% chance to gain $1 while the other player loses $1 and vice-versa. After making their choice, participants indicated their age, gender, and race/ethnicity.

**Results**

We predicted that even though they would be matched with another player who made the same decision as themselves (and therefore would not be responsible for that player’s outcomes), participants would prefer to separate the two players’ payoffs from each other. Indeed, although both games offered the same likelihood of gaining or losing, and although each player was responsible for their own decision of which game to play, participants overwhelmingly chose the non-zero-sum game (72%, n = 73) over the zero-sum game (28%, n = 29), *χ^2^*(1,99) = 19.62, *p* < .001. Thus, participants again exhibited zero-sum aversion, opting to separate their and others’ gains and losses.

**Study S3E: Ruling out reluctance to interdependence**

Study S3E examines whether zero-sum aversion reflects a general reluctance to having linked outcomes or a specific aversion to *inversely* linked outcomes (linking one’s gains to others’ losses and vice-versa). That is, people may be reluctant to have their outcomes interdependent on others’ outcomes and vice-versa, regardless of whether this interdependence is zero-sum or non-zero-sum. Indeed, Wiltermuth & Cohen (2014) suggest that, because they worry about letting others down, guilt-prone individuals often avoid interdependence payments schemes, where their outcomes are positively correlated with others’ outcomes. To examine this, participants chose between two games—one where payoffs are inversely linked (i.e., zero-sum) and one where payoffs are *positively* linked (i.e., *non*-zero-sum). If zero-sum aversion accounts for their preferences, then participants should opt for the non-zero-sum game even though it (positively) links the players’ payoffs.

**Method**

**Participants**. One hundred six U.S. residents were recruited from Amazon’s Mechanical Turk (*M_age_* = 39.49; 43 females, 56 males, 7 other/did not specify; 78.1% White, 5.7% Black, 3.8% Hispanic, 6.7% East Asian, 3.8% South Asian, 1% Indigenous/Native American, 1% Other), giving us 80% power to detect ratios as small as 1.46 in a one-sample chi-square design.

**Materials and Procedure**. Participants chose between two games: a *non-zero-sum* game and a *zero-sum* game. The *zero-sum* game was a combined coin-flip that gave participants 50% chance of gaining $1 while the other player loses $1 and 50% chance of losing $1 while the other player gains $1. The *non-zero-sum* game also involved a combined coin-flip, but one where outcomes were *positively* linked, giving participants 50% chance of gaining $1 while the other player also gains $1 (i.e., joint gains) and 50% chance of losing $1 while the other player also loses $1 (i.e., joint losses). Thus, although the payoffs in both games were linked, they differed in whether one player’s gains would be offset by the other player’s losses (Figure S6). After making their choice, participants indicated their age, gender, and race/ethnicity.

**Results**

If participants are specifically averse to *inversely* linked outcomes (rather than generally averse to linking their and others’ outcomes), they should exhibit zero-sum aversion even when the non-zero-sum game involves a combined coin-flip. Indeed, although payoffs were linked in both games, participants were significantly more likely to choose the non-zero-sum game (87%, n = 92) than the zero-sum game (13%, n = 14), *χ^2^*(1,105) = 64.20, *p* < .001, showing a specific reluctance to *inversely* linking their and others’ outcomes (i.e., zero-sum aversion).

**Study S3F: Ruling out inequity aversion**

Study S3F examines whether zero-sum aversion is due to the inequity inherent to zero-sum situations. In zero-sum situations, a person’s gains are offset by others’ losses, creating either disadvantageous inequity (e.g., losing while others gain) or advantageous inequity (e.g., gaining while others lose). Since non-zero-sum situations can sometimes create more equitable distributions of resources (i.e., joint gains or losses), people may exhibit zero-sum aversion because they dislike inequity (e.g., Dawes, Fowler, Johnson, McElreat, & Smirnov, 2007; Fehr & Schmidt, 1999). To examine this, participants chose between two games in which the non-zero-sum option created higher inequity than the zero-sum option. If participants are truly averse to zero-sum situations, then they should choose the non-zero-sum game even when it creates higher inequity between the players.

**Method**

**Participants**. One hundred one U.S. residents were recruited from Amazon’s Mechanical Turk (*M_age_* = 40.58; 51 females, 50 males; 77.7% White, 5.7% Black, 1% Hispanic, 11.7% East Asian, 3.9% South Asian, 1% Indigenous/Native American, 1% Middle Easter/Arabic), giving us 80% power to detect ratios as small as 1.47 in a one-sample chi-square design.

**Materials and Procedure**. Participants chose between two games: a *non-zero-sum* game with high inequity or a *zero-sum* game with lower inequity. The *zero-sum* game involved a combined coin-flip that guaranteed exactly $2 difference in payoffs, giving each player 50% chance of gaining $1 while the other player loses $1 and 50% chance of losing $1 while the other player gains $1. The *non-zero-sum* game involved separate coin-flips that independently determine each player’s outcomes. These coin-flips provided different payoffs to each player, giving participants 50% chance of gaining or losing $3 and the other player 50% chance of gaining or losing $1. Thus, although the non-zero-sum game still offered potential for joint gains and losses, it also guaranteed that the difference in payoffs would be *at least* $2 (if participants won $3 and the other player only won $1 or if participants lost $3 and the other player only lost $1), and potentially as high as $4 (if participants won $3 and the other player lost $1 or if they lost $3 and the other player won $1) (Figure S6). After making their choice, participants indicated their age, gender, and race/ethnicity.

**Results**

If participants are averse to zero-sum outcomes rather than the inequity inherent to them, then they should choose the non-zero-sum game even when it potentially creates higher inequity. Indeed, although both games create inequity, participants significantly preferred the non-zero-sum game (72%, n = 73) than the zero-sum game (28%, n = 28), *χ^2^*(1,101) = 20.77, *p* < .001. Although inequity aversion may play some role in zero-sum aversion, it does not seem to be a necessary condition for it. And, since participants in Study S3E who chose the non-zero-sum game risked losing $3 (vs. $1 in the zero-sum game) and since losses loom larger than gains (Tversky & Kahneman, 1981), the fact that participants exhibit zero-sum aversion in this study is especially revealing.

**Study S3G: Ruling out affective expectations**

Study S3G examines whether zero-sum aversion is due to the expectation that winning and losing both feel worse in zero-sum situations. Although mixed outcomes (i.e., gaining while others lose or vice-versa) exist in both situations, they may feel worse in zero-sum situations than in non-zero-sum situations. Losing might be more painful and gaining less enjoyable when each person’s outcomes are dependent on (rather than independent from) others’ outcomes. To examine this, we asked participants how they would feel about gaining and losing in either a zero-sum or a non-zero-sum game.

**Method**

**Participants**. One hundred one U.S. residents were recruited from Amazon’s Mechanical Turk (*M_age_* = 39.81; 58 females, 43 males; 78% White, 7.3% Black, 2.9% Hispanic, 5.5% East Asian, 2.8% South Asian, 1% Indigenous/Native American), giving us 80% power to detect small effects (*d* = 0.28) in a one-sample t-test and ratios as small as 1.46 in a chi-square test.

**Materials and Procedure**. Participants read about a *non-zero-sum* game (independently giving them and another player 50% chance of gaining or losing $1) and a *zero-sum* game (giving each player 50% chance of gaining at the other player’s expense). Following, participants reported, in counterbalanced order, which game would make them feel better (a) if they gained while the other player lost and (b) if they lost while the other player gained (*1-I would feel better in the two separate coin-flips, 4-I would feel equally good or bad in both, 7-I would feel better in the one combined coin-flip*). Finally, participants indicated their preference between the games (*1- I would much prefer the two separate coin-flips, 3- No preference, 5- I would much prefer the one combined coin-flip*) and reported their age, gender, and race/ethnicity.

**Results**

First, we examined whether participants expected to feel worse about gaining/losing in the zero-sum game or the non-zero-sum game. Participants expected to feel equally good or bad about gaining while the other player lost regardless of the game they played (M = 3.83, SD = 2.02, 95% CI[3.43,4.23], one sample t-test relative to the midpoint (4) *t*(100) = 0.83, *p* = .40, *d* = 0.08). Similarly, they expected to feel equally good or bad about losing while the other player gained regardless of the type of game (M = 3.70, SD = 1.68, 95% CI[3.35,4.05], one sample t-test relative to the midpoint (4) *t*(100) = 1.68, *p* = .096, *d* = 0.18).

Despite expecting to feel the same about their outcomes, participants overwhelmingly preferred to play the non-zero-sum game (M = 2.56, SD = 1.39, 95% CI[2.29,2.84], one sample t-test relative to the midpoint (3) *t*(100) = 3.15, *p* = .002, *d* = 0.32). Of those who showed a preference for one game over the other, 67% (n = 56) preferred the non-zero-sum game but only 33% preferred the zero-sum game (n = 27), *χ^2^*(1, 82) = 5.26, *p* = .021. Thus, although caution should be taken when interpreting null results, our findings suggest that expectations of how winning and losing feels in zero-sum situations do not seem to drive zero-sum aversion.

**Study S3H: Further ruling out affective expectations**

Study S3G examined whether participants’ predictions regarding how they would feel about winning or losing account for their zero-sum aversion. Since participants may feel more negative when others gain at their expense and less positive when they gain at others’ expense, they may opt to maximize their emotional reaction by separating their and others’ gains and losses (i.e., non-zero-sum). To examine this, we asked participants in Study S3H to choose between a zero-sum coin-flip and a non-zero-sum coin-flip for two other people. If participants are averse to zero-sum situations simply because they try to maximize their emotional reactions, then choosing for two other people should eliminate this bias.

**Method**

**Participants**. One hundred eleven U.S. residents were recruited from Amazon’s Mechanical Turk (*M_age_* = 39.25; 48 females, 53 males, 10 other/did not specify; 80.9% White, 8.6% Black, 2.9% Hispanic, 6.7% East Asian, 1% South Asian). This sample size allows us to detect a ratio as small as 1.45 in a one-sample chi-square design with 80% power.

**Materials and Procedure**. Participants imagined that they would be flipping a coin for two people other than themselves and indicated which of these options they would choose to play: a *non-zero-sum* option or a *zero-sum* option. The *non-zero-sum* option involved two separate coin-flips, giving each of the people 50% chance of winning $1 and 50% chance of losing $1, and guarantees that their outcomes would be independent from each other. The *zero-sum* option involved one combined coin-flip which gave each person 50% chance of winning $1 while the other person loses $1, and therefore did not offer an opportunity for joint gains or losses. After making their choice, participants were asked to explain their decision and indicated their age, gender, and race/ethnicity.

**Results**

If participants in Studies S3A-S3G were averse to the zero-sum options because the pain of losing is stronger when others’ gain at one’s expense and the joy of gaining is weaker when others lose as a consequence, than they should be indifferent between the two options that determine *other* *people’s* gains and losses. In contrast, if participants are averse to zero-sum situations because they see them as unfair, they should exhibit zero-sum aversion even when both options determine others’ outcomes. Indeed, despite the fact that their own outcomes would not be affected by their choices, participants were significantly more likely to choose the non-zero-sum, two separate coin-flips (59.5%, n = 66) than the zero-sum, one combined coin-flip (40.5%, n = 45), *χ^2^*(1, 110) = 3.99, *p* = .046. Thus, although participants were somewhat more willing to choose the zero-sum option than previous studies, they still exhibited a significant zero-sum aversion, opting to separate each person’s gains and losses from the other person’s outcomes.

**Supplemental** **Study S4**

Study S4 provides initial evidence that the expectation of conflict in zero-sum situations is a driving force behind zero-sum aversion. Before indicating their preference between a zero-sum and a non-zero-sum game, participants reported how much tension, animosity, and conflict each game would create between the players. Although both are games of chance, we predicted that participants would view the zero-sum game as more conflict-prone and, consequently, would be averse to playing it.

**Method**

**Participants**. One hundred one U.S. residents were recruited from Amazon’s Mechanical Turk (*M_age_* = 41.19; 44 females, 56 males, 1 Nonbinary; 78.4% White, 4.9% Black, 4.9% Latin/Hispanic, 8.8% East Asian, 1% South Asian, 1% Indigenous/Native American, 1% Middle Eastern/Arabic), giving us 80% power to detect small effects (*dz* = 0.28) in a matched-pairs test.

**Materials and Procedure.** Participants viewed, in counterbalanced order, the zero-sum and non-zero-sum games from Study 2A. For each game, participants indicated how much “harmful conflict,” “friction,” and “animosity” it would create between the players, how much it would feel like they “are pitted against each other,” and how much it would “put tension” on their potential relationship (*1-Not at all; 7-Very much so*;*α*_zero-sum_ = 0.94, *α*_non-zero-sum_ = 0.95). Following, participants indicated which game they would prefer to play (*1- I would much prefer the two separate coin-flips, 3- No preference, 5- I would much prefer the one combined coin-flip*), and reported their age, gender, and race/ethnicity.

**Results**

Replicating Studies 1-3, participants exhibited significant zero-sum aversion, preferring the non-zero-sum game over the zero-sum game (M = 2.09, SD = 1.30, 95% CI[1.83,2.35]), one sample t-test relative to the midpoint (3) *t*(100) = 7.02, *p* < .001, *d* = 0.70. Of those who showed a preference for one game over the other, 79.5% (n = 66) preferred the non-zero-sum game but only 20.5% preferred the zero-sum game (n = 17), *χ^2^*(1, 82) = 30.90, *p* < .001.

Next, we examined participants’ expectations regarding conflict in each game. Although both games were determined by chance, participants believed that the zero-sum game would create more conflict and animosity between them and the other player (M = 3.81, SD = 1.72, 95% CI [3.47,4.15]) than the non-zero-sum game (M = 2.40, SD = 1.56, 95% CI[2.09,2.71]), matched-pairs *t*(100) = 7.49, *p* < .001, *dz* = 0.74.

Finally, we examined whether viewing zero-sum situations as more conflict-prone predicted zero-sum aversion. A multiple regression analysis revealed that participants’ aversion to the zero-sum game was significantly predicted by their belief that it would create hostility and conflict (*β* = 0.26, *t* = 3.75, *p* < .001) as well as by their belief that the *non*-zero-sum game would not be as conflict-prone (*β* = -0.45, *t* = 3.75, *p* < .001). The more participants expected the zero-sum game to create tension and animosity between the players, and the less they expected the non-zero-sum game to do so, the more they exhibited zero-sum aversion, choosing to separate their and the other player’s outcomes. Thus, expectation of conflict predicted zero-sum aversion.

*ß =* 1.16

*p < .*001

*ß =* 0.57

*p < .*001

*ß =* 0.04*, p = .*88

*(ß* = 0.70, *p =* .004)

Fear of conflict

Type of negotiation (zero-sum vs. non-zero-sum)

Zero-sum aversion

**Figure S1***.* The mediating role of perceived conflict on the relationship between type of negotiation (zero-sum vs. non-zero-sum) and zero-sum aversion (Study 4B).

*ß =* 2.38

*p < .*001

*ß =* 0.64

*p < .*001

Fear of conflict

Review type (zero-sum vs. non-zero-sum)

Zero-sum aversion

*ß =* 0.56*, p = .*07

*(ß* = 2.09, *p <* .001)

**Figure S2***.* The mediating role of perceived conflict on the relationship between type of review process (zero-sum vs. non-zero-sum) and zero-sum aversion (Study 4C).


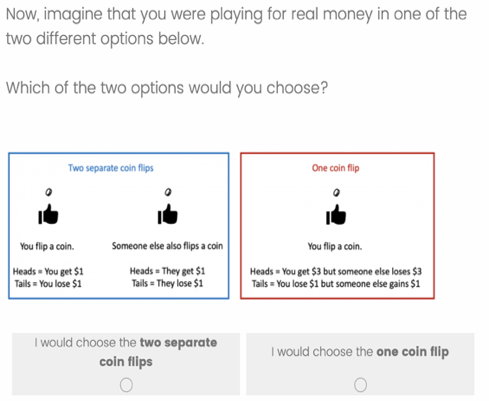


End of study


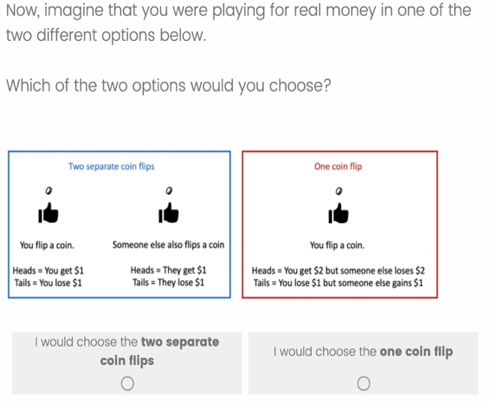


End of study


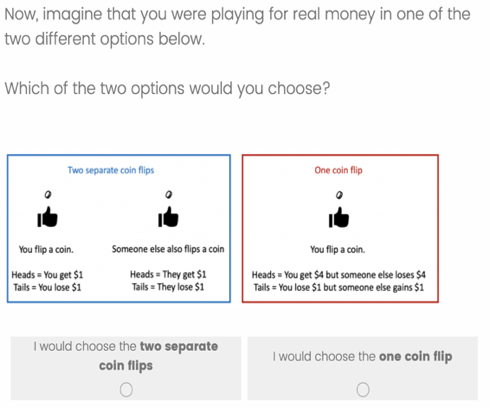


End of study


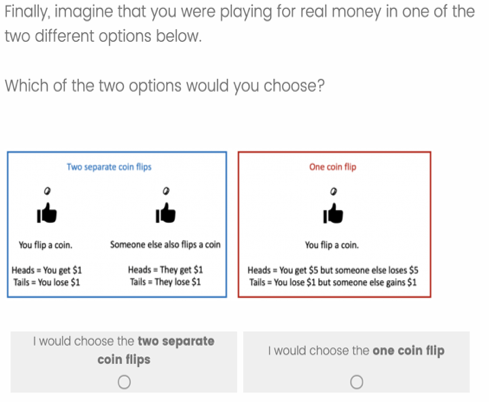


End of study


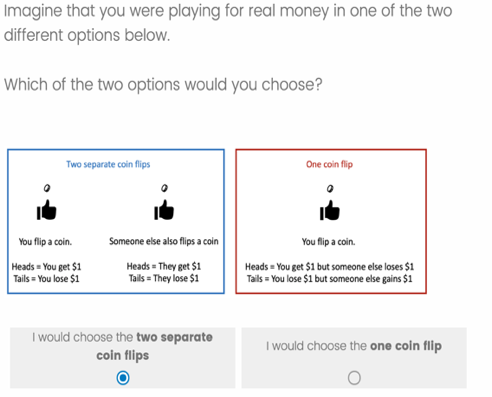


End of study

Choice #1

Choice #2

Choice #3

Choice #4

Choice #5

**Figure S3.** Research design of Study S1. At each stage, participants who chose the non-zero-sum option (two separate coin-flips, left side) continued to the next choice, where the expected value of the zero-sum option increased in $1 increments. Participants who chose the zero-sum option (one coin-flip, right side) ended the study

**Figure S4**. The cumulative likelihood of choosing the zero-sum option at each incremental $1 payoff increase of the zero-sum option (Study S1).

**Figure S5.** The cumulative likelihood of choosing the zero-sum option at each incremental 10% likelihood increase of the zero-sum option (Study S2)

| 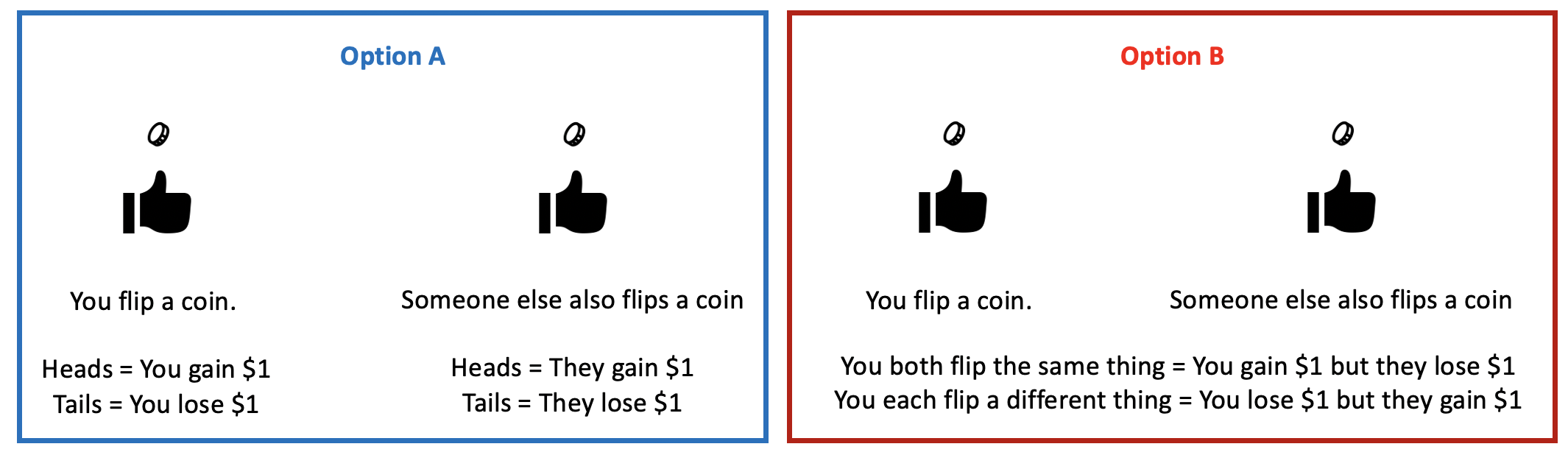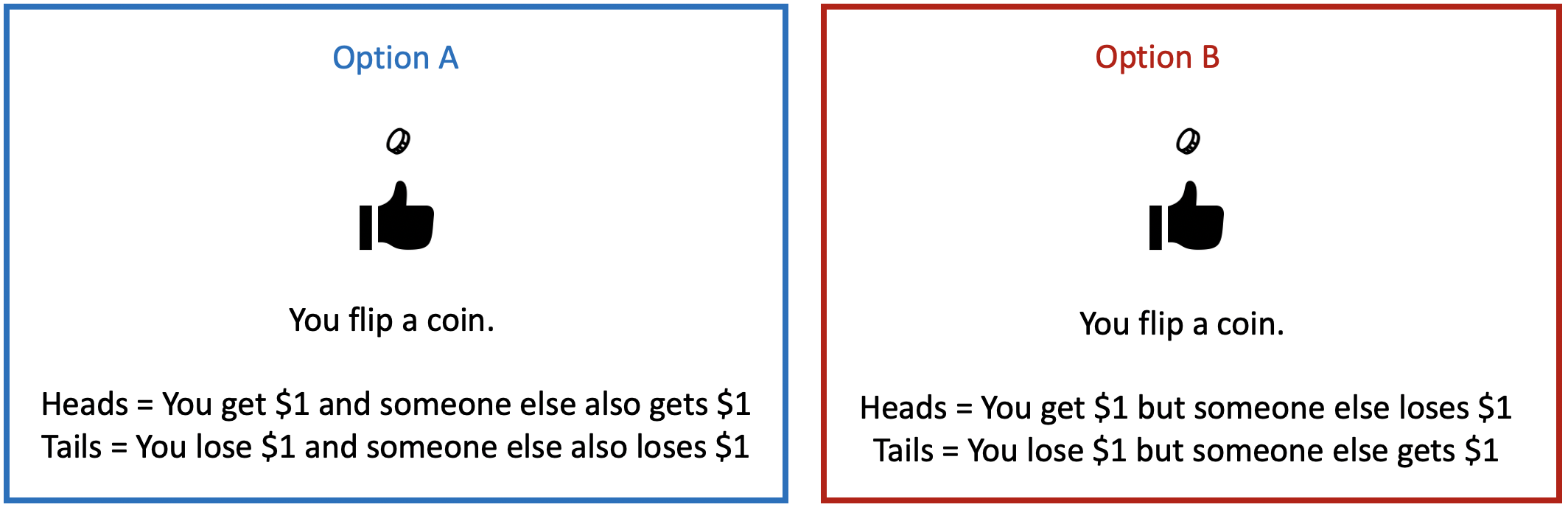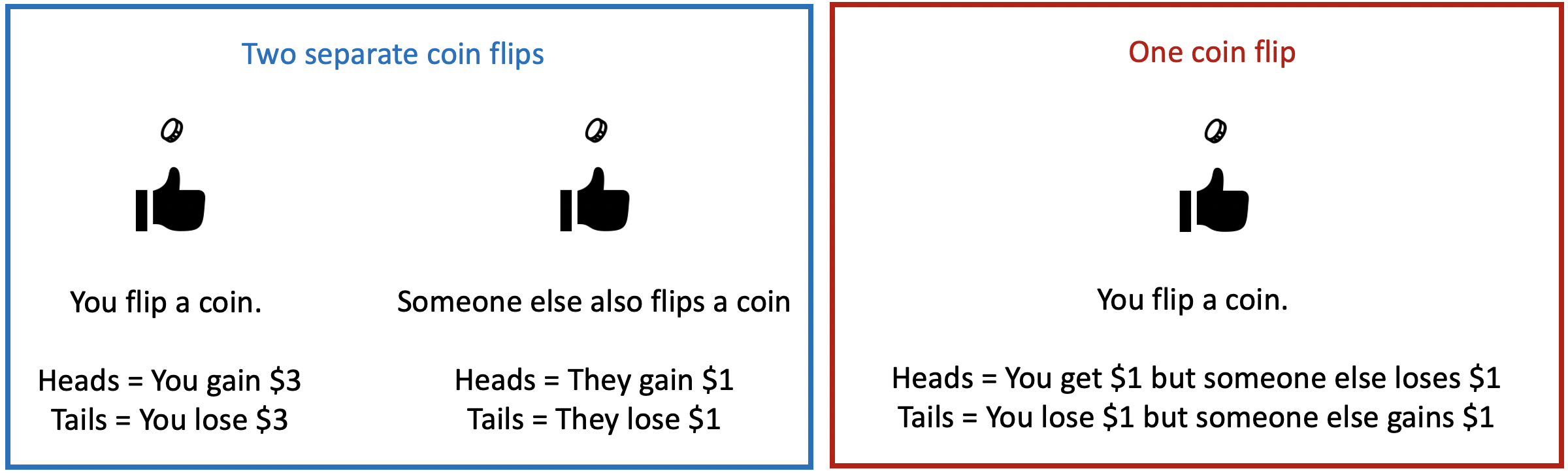 |
| --- |

**Figure S6.** The non-zero-sum (left) and zero-sum (right) options in Studies S3A (top), S3E (middle), and S3F (bottom).

**Pre-registration links**

Study 2C: https://aspredicted.org/7GJ_W8R

Study 3A: https://aspredicted.org/OYC_WIB

Study 3B: https://aspredicted.org/NRG_S37

Study 4B: https://aspredicted.org/UQJ_HYK

Study 4C: https://aspredicted.org/S7H_QQ6
